# Structure and Wiring Optimized TT/MT Double‐Helical Fiber Sensors: Fabrication and Applications in Human Motion Monitoring and Gesture Recognition

**DOI:** 10.1002/advs.202416564

**Published:** 2025-02-04

**Authors:** Ziwei Chen, Daoxiong Qian, Dandan Xie, Chunxia Gao, Jian Shi, Hideaki Morikawa, Chunhong Zhu

**Affiliations:** ^1^ Graduate School of Medicine, Science and Technology Shinshu University 3‐15‐1 Tokida Ueda Nagano 3868567 Japan; ^2^ Institute for Fiber Engineering and Science (IFES) Shinshu University 3‐15‐1 Tokida Ueda Nagano 3868567 Japan; ^3^ School of Chemistry and Chemical Engineering Yangzhou University Yangzhou 225002 China; ^4^ Faculty of Textile Science and Technology Shinshu University 3‐15‐1 Tokida Ueda Nagano 3868567 Japan

**Keywords:** coaxial wet‐spinning, double‐helical fiber, flexible sensor, gesture recognition, human motion monitoring

## Abstract

A fibrous flexible sensor, with its small size, minimally burdens the human body, ranking among the most user‐friendly flexible sensors. However, its application is often limited by damage caused by electrode movement, as flexible sensors are typically attached to joints, which can be greatly alleviated by placing the two electrodes on the same side. Inspired by the hydrogen bonds in the double‐helical structure of DNA, the double‐helical electrode design is commonly found and applied in fiber‐based batteries and supercapacitors into fibrous flexible sensors through coaxial wet‐spinning and further treatment. The double helical sensor exhibits high strength and maintains stable operation and is prepared under over 300% strain with gauge factors (GF) of 0.9, 39.5, and 349, respectively, in its working ranges. This unique single‐sided electrode structure also enabled applications such as water flow sensing. The sensor into a smart glove capable of real‐time is further integrated, five‐channel finger motion detection, and used a convolutional neural network (CNN)‐based machine learning algorithm to achieve 98.8% accuracy in recognizing six common gestures. This study provides a novel approach to optimize the electrode distribution in fiber‐based flexible sensors through an internally encapsulated double‐helical structure, making a significant contribution to the field of flexible sensing.

## Graphical abstract

By precisely tuning a coaxial wet‐spinning process, the delicate, fluffy core–shell fibers have been crafted. When two of these fibers are twisted together and subjected to controlled heat treatment, their surfaces seamlessly bond without damage, yielding a perfectly merged double‐helical fiber with an almost circular cross‐section. This innovative structure forms the single‐sided and dual‐electrode fiber‐based flexible sensors, offering unique applications.

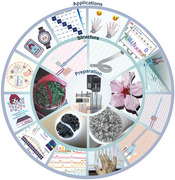

## 1 Introduction

Flexible sensors, currently a hot research topic,^[^ ^1^, ^2^, ^3^, ^4^ ^]^ demand particular attention in their design and structure as they come into direct contact with the user's body.^[^ ^5^, ^6^, ^7^, ^8^ ^]^ Therefore, it is crucial to ensure that their shape and structure are user‐friendly. From a shape perspective, flexible sensors can be block‐like 3D,^[^ ^9^, ^10^, ^11^, ^12^ ^]^ film or fabric‐like 2D,^[^ ^13^, ^14^, ^15^ ^]^ or fiber‐like 1D.^[^ ^16^, ^17^, ^18^ ^]^ Fiber‐like structures are particularly user‐friendly due to their minimal volume, reducing the burden during wear.^[^ ^19^, ^20^ ^]^ However, they also have significant drawbacks, especially their susceptibility to damage. For example, when fiber ends are connected to electrodes on human joints, due to the movement of the joints — where at least one side frequently moves with the joint — the weight of the electrode's connecting wires can continually tug on the electrode due to inertia during movement, causing poor contact or even damage. This issue is often encountered in practical applications, but it is rarely mentioned in academic papers.

Positioning both electrodes on the same side of the sensor and placing them in an area with a relatively small range of motion can significantly reduce the risk of sensor damage caused by joint movement. Effective electrode design is critical to the lifetime and performance of sensor components, as seen in the commonly used interdigital electrodes in pressure or humidity sensors,^[^ ^21^, ^22^, ^23^ ^]^ which are designed with both connection electrodes on one side at a certain distance from the sensing material for better sensing quality. However, addressing electrode issues in 1D fiber strain sensors is often challenging and overlooked. Inspired by the common double‐helical structure of fiber batteries^[^ ^24^ ^]^ and supercapacitors,^[^ ^25^, ^26^ ^]^ applying this structure to flexible sensors to create a same‐side dual‐electrode strain sensor has the potential to address the inconvenience and damage caused by electrode pulling at joint areas.

Fabricating flexible sensors with a same‐side dual‐electrode and a double‐helical structure, similar to those used in fiber batteries and supercapacitors, is time‐consuming and expensive, significantly increasing the volume and cost of the sensor. A new approach is needed. Inspired by the double‐helical structure of DNA, using polymers that bond tightly internally after twisting, like the hydrogen bonds of DNA, could be a simpler and more efficient encapsulation method. Coaxial wet‐spinning, a recent research hotspot,^[^ ^27^, ^28^, ^29^, ^30^, ^31^, ^32^ ^]^ allows the continuous production of multilayer fibers with a core‐shell structure. By designing the core as a fluffy conductive layer and the shell as an insulating layer that bonds during heat treatment, the resulting coaxial fibers can be twisted into a double‐helical structure. After heat treatment, by connecting two electrodes on one end and using conductive silver glue at the other ends, a simple, internally encapsulated flexible strain sensor can be created, which largely solves existing application problems in flexible sensors.

Our plan is to use thermoplastic polyurethane (TPU), which is widely used as a base material,^[^ ^33^, ^34^, ^35^ ^]^ and multi‐walled carbon nanotube (MWCNT), which is currently widely used as a conductive filler,^[^ ^34^, ^36^, ^37^, ^38^ ^]^ to fabricate a coaxial core‐shell fiber with a thin insulating bonding layer as the outer shell and a fluffy conductive layer as the core by coaxial wet‐spinning. By twisting two such fibers into a double‐helical structure that resembles a yin‐yang or “tai chi” structure when viewed in cross‐section, and selecting an appropriate heat treatment to fuse the shell layers of the fibers, we aim to maximize the durability, mechanical strength, and sensing performance of the entire fiber‐like flexible sensor. To achieve this goal, we use a slow coaxial spinning method with different speeds for the core (3 mL h^−1^) and the shell (0.75 mL h^−1^) and improve the wet‐spinning process by incorporating nano‐TiO_2_ into the mixture for the sheath layer. The TiO_2_ nanoparticles increase the density of the mixture and molding speed, resulting in thicker, fluffier fibers. Moreover, experimental results have shown that incorporating nano‐TiO_2_ changes the surface structure of the fiber. This innovative method produces a special fiber with a core‐shell structure, facilitating the manufacture of fiber sensors with a double‐helical structure and same‐side dual‐electrode, which is suitable for practical applications such as motion and fluid flow sensing. Compared to the traditional spinning method combined with a spinneret design, the most significant advantage of our innovative approach lies in the natural formation of positive and negative terminals at both ends of the two TT/MT fibers during the twisting process. This significantly reduces the risk of short circuits and disconnections, eliminating the need to weld two electrodes within the delicate double‐core fibers. In addition, this sensor structure can be used to produce smart gloves with electrodes only on the back of the hand, enabling real‐time multi‐channel finger movement monitoring and gesture recognition via convolutional neural network (CNN)‐based machine learning. This novel double‐helical fiber sensor has substantial practical value for future applications.

## 2 Experimental Section

### 2.1 Materials

TPU was provided by Covestro AG, Germany (product code 385SX, the infrared spectrum is shown in Figure S1 (Supporting Information). N,N‐Dimethylformamide (DMF) was sourced from Fujifilm Wako Pure Chemical Corporation, Japan. MWCNTs, with lengths of 3–12 micrometers and inner diameters of 3–5 nanometers, were acquired from Shenzhen Suiheng Technology Co., Ltd., China. Nano‐sized TiO_2_, in the form of nanopowder with a primary particle size of 21 nm, was supplied by Sigma‐Aldrich, USA. All chemicals were of analytical grade and were used as received. Deionized water was used in all experiments.

### 2.2 Slurry Configuration

Core Part: Add 8.6 g of DMF into a thoroughly clean and dry container, then add 0.4 g of MWCNT. After stirring for 10 min, place the mixture in an ultrasonic device and treat it with ultrasound for 1 h. Then, add 1 g of TPU particles and heat while stirring at 90 °C for 10 h in a dry environment to obtain a black slurry.

Shell Part: Add 7.8 g of DMF into a thoroughly clean and dry container, then add 0.2 g of nano‐TiO_2_. After stirring for 10 min, place the mixture in an ultrasonic device and treat it with ultrasound for 1 h. Then, add 2 g of TPU particles and heat while stirring at 90 °C for 10 h in a dry environment to obtain a milky white slurry.

### 2.3 Wet‐Spinning Process

Two spinning injection pumps were set up horizontally (for the shell) and vertically (for the core), respectively, for coaxial wet‐spinning to fabricate core‐shell structure fibers. The coaxial needle used has an outer diameter of 17 gauge and an inner diameter of 22 gauge. The needle was immersed in a coagulation bath containing 500 mL of a 20% ethanol aqueous solution. The core layer solution and shell layer solution were both loaded into syringes with a 2 cm diameter, with the shell injection pump (horizontal) set at a speed of 0.75 mL h^−1^ and the core injection pump (vertical) set at 3 mL h^−1^, ensuring the flow rate ratio of 1:4 at the needle tip, to produce unique structural features of the fiber (the specific reasons for the various parameter settings are explained in detail in the Results and Discussion section). After spinning, the fibers were soaked in the coagulation bath for at least 3 h to ensure complete solidification. The fibers were then soaked in fresh water for a further 12 h or more to completely remove the DMF. Finally, the fibers were dried at 50 °C, resulting in TT/MT fibers with a thin insulating adhesive outer layer and a fluffy conductive inner layer with a coaxial core‐shell fiber structure.

To simplify the description, it was referred to the fibers with TiO_2_ and TPU in the outer layer and MWCNT and TPU in the inner layer as TT/MT fibers. Similarly, for comparison, fibers prepared under the same conditions without the addition of TiO_2_ in the outer layer will be referred to as T/MT fibers.

### 2.4 Characterizations and Measurements

The surface morphology and structural features were observed using Scanning Electron Microscopy (SEM, JSM‐6010LA, JEOL, Japan) and Field Emission Scanning Electron Microscopy (FESEM, JSM‐IT800SHL, JEOL, Japan). EDS analysis data were also obtained using the JEOL EDS system of Scanning Electron Microscopy (SEM, JSM‐6010LA, JEOL, Japan). The optical microscopy of the fibers was conducted using a high‐speed microscope (VW‐9000, Keyence, Japan). The infrared spectra of the polyurethane used were obtained with a Fourier Transform Infrared Spectrometer (FT‐IR, IR‐Prestage21, Shimadzu Corporation, Japan). The mechanical properties were evaluated using a tensile tester (MCT‐2150, A&D, Japan). Furthermore, the electrical properties of the double‐helical sensors prepared by TT/MT fibers were evaluated using the tensile tester in conjunction with an electrometer (Keithley‐6514, Keithley Instruments, USA). The same electrostatic meter was also used in practical application tests such as human movement. An Arduino Uno microcontroller was used as the connection device for testing smart gloves. The relationship between the mass and temperature of TT/MT fibers was determined using Thermogravimetric analysis (TG, TG‐8120, Thermo Plus, USA) with a heating rate of 10 °C min^−1^.

## 3 Results and Discussion

**Figure 1:**
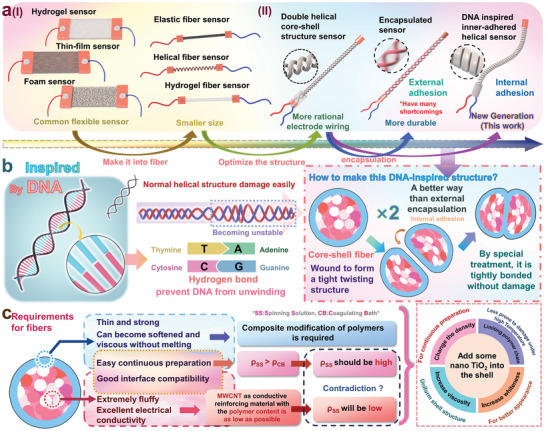
Evolution of flexible sensors and design concept of inner‐adhesive and double‐helical sensors: a) The demonstration of flexible sensors with various structures. b) The inspiration that the DNA structure brings to the preparation of the internally bonded double‐helical structure. c) The requirements for and solutions to the preparation of the internally bonded double‐helical sensors in wet‐spinning.

**Figure** **1** illustrates the design and fabrication concept of a TT/MT fiber sensor with a double‐helical structure, where the electrodes are on the same side. As shown in Figure 1a, from the perspective of user‐friendly practical applications, the shape of flexible sensors can change and evolve. The most basic and common form is a block or film‐type flexible sensor, where these sensors benefit from a stable working signal due to their extensive internal conductive networks. However, their modulus is typically much higher than that of human skin, which can cause discomfort when worn. With the trend toward miniaturization in flexible devices, fiber‐shaped sensors have gained attention. However, as mentioned above, when detecting human motion, movement at the joints can pull on the wires, leading to electrode displacement and sensor damage. By positioning both electrodes on the same side of the sensor and placing this side on areas of the skin with minimal movement (e.g., the back of the hand during finger movement where movement is less pronounced compared to the fingertips), such problems can be significantly mitigated. Accordingly, three viable solutions are proposed in Figure 1a(II): utilizing two helical fibers to form a circuit (1), encapsulating these fibers with a polymer after circuit formation (2), or twisting two specially structured core‐sheath fibers into a double‐helical with internal fixation (internal encapsulation) (3). The latter approach avoids the problems of damage susceptibility or complex fabrication processes associated with the first two approaches. Thus, our plan involves the internal fixation of a double‐helical structure using specially structured core‐sheath fibers (internal encapsulation) to fabricate the coaxial electrode, as well as double‐helical TT/MT fiber sensor.

As shown in Figure 1b, the hydrogen bonds within DNA significantly stabilize its structure, and prevent it from unraveling. When two fluffy, twisted fibers are compressed, they have extensive surface contact and internal stress. After thermal treatment, the internal surfaces are fused together, resulting in a robust, durable double‐helical fiber sensor that resists unraveling. This places specific demands on the fibers' properties used in the fabrication. As indicated in Figure 1c, the fiber skin must be sufficiently thin yet durable and able to fuse without damage under certain conditions, while the fiber interior must be fluffy and conductive. In wet‐spinning, more porous, fluffier fibers can be achieved by reducing the polymer concentration in the dope. However, too low polymer concentrations can hinder the wet‐spinning process. To meet these requirements, we propose incorporating TiO_2_ into the spinning dope of the shell, which has four advantages: it increases the density of the dope, facilitating wet‐spinning (I); it makes fiber surface less prone to damage under high temperature (II); it enhances the viscosity of the spinning solution, leading to more uniform fiber skins (III); and it improves the aesthetic appearance of the sensor by rendering it whiter (IV).

**Figure 2:**
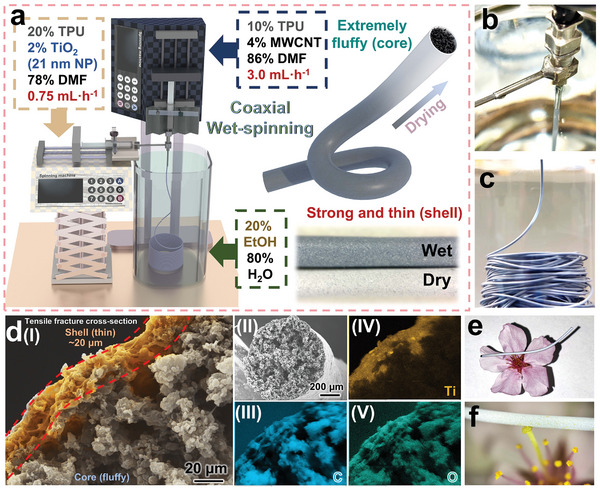
Preparation of TT/MT fluffy core‐shell fibers: a) Schematic diagram of wet‐spinning and the proportion of slurry. b) Photo of the needle for wet‐spinning. c) Photo of fibers during the wet‐spinning process. d) Cross‐sectional SEM image of TT/MT fluffy fibers and EDS map of the external structure of TT/MT fluffy fibers. e,f) Display of the lightweight TT/MT fibers.

**Figure** **2** illustrates the fabrication and morphology of the TT/MT fibers for the preparation of double‐helical flexible sensors. Figure 2a clearly presents a schematic diagram of the TT/MT fiber production, highlighting the selection of various parameters. The slurry of the shell layer contains a higher concentration of thermoplastic polyurethane (TPU) at 20 wt.%, with the addition of 2 wt.% nano‐TiO_2_ (21 nm TiO_2_ nanoparticles, 21 nm NP for short), which ensures a compact shell of the TT/MT fibers. In contrast, the core layer slurry contains a lower TPU concentration at 10%, enriched with 4% multi‐walled carbon nanotubes (MWCNTs) to achieve a fluffy internal structure with improved conductivity. Additionally, a coagulation bath consisting of a 20% ethanol aqueous solution was selected based on density considerations. For successful wet‐spinning, the density of the spinning slurry must exceed that of the coagulation bath; otherwise, the slurry would float to the surface and potentially clog the spinneret. At room temperature, the relative density of DMF is ≈0.95, which increases slightly as the TPU dissolves and the MWCNTs are mixed. However, to maintain a fluffy structure, the slurry concentration used in this work is low. Even with the inclusion of nano‐TiO_2_ to increase relative density, spinning in pure water is challenging. Therefore, we choose a 20% ethanol solution with a relative density of ≈0.97. The shell and core structures were spun at rates of 0.75 and 3 mL h^−1^, respectively, to achieve a thin‐skinned, core‐rich fiber structure which is easy to be compressed when twisted. Initially, the fibers appear a pale blue‐black during spinning and turn white upon drying, as shown in Figure 2b,c, which depicts the continuous and reliable spinning process. A video of this process is also presented in Movie S1 (Supporting Information). The infrared spectra of TT/MT fibers are shown in Figure S2 (Supporting Information), Figure 2d shows a cross‐sectional observation of a TT/MT fiber via energy‐dispersive X‐ray spectroscopy (EDS), where the distribution of Ti elements indicates that the thickness of shell structure is ≈20 µm. Elemental mapping of Ti, C, and O reveals a continuous, compact layer of Ti (from the added nano‐TiO_2_) in the fiber's skin, while the inner layers exhibit a non‐compact, spongy and fluffy structure rich in C and O, characterizing the fibers with a dense exterior and a porous and compressible interior. As demonstrated in Figure 2e,f, the fibers are extremely lightweight and fluffy, as a single cherry blossom bud can withstand TT/MT fibers longer than its own petals.

**Figure 3:**
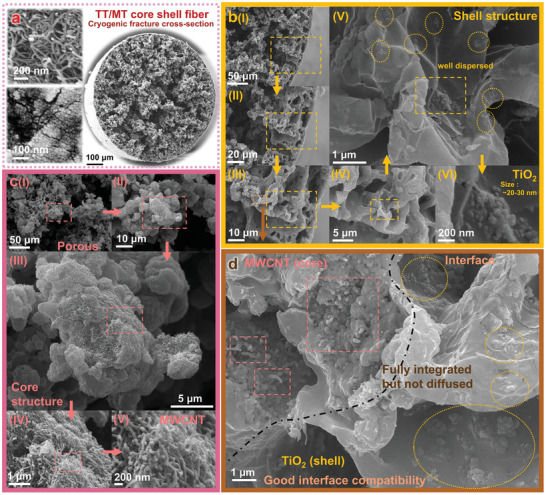
FESEM characterization of TT/MT fluffy core‐shell fiber structure: a) Cross‐sectional images of raw materials and fiber. b) Structural characterization of the epidermis of TT/MT fluffy core‐shell fibers. c) Structural characterization of the internal TT/MT fluffy core‐shell fibers. d) Structural characterization of the interface between the core and the shell of TT/MT fluffy fibers.

In **Figure** **3**, to further analyze the TT/MT fibers, we used liquid nitrogen to fracture the fibers, followed by gold sputtering for 60 s, and subsequently observed the cross‐sections using FESEM. First, Figure 3a presents the SEM images of the raw materials, MWCNT and TiO_2_, used in the preparation of TT/MT fibers (for more detailed data, refer to Figure S3, Supporting Information), together with the overall SEM image of the fractured cross‐section (brittle fracture using liquid nitrogen). Figure 3b focuses on a more detailed examination of the outer surface of the TT/MT fiber. As shown in Figure 3b(I),(II), the outer surface of the fiber is smooth and free from irregularities, ensuring surface uniformity. Figure 3b(III),(IV) show that, although the outer layer still contains large pores of ≈10 µm and smaller pores ≈1 µm, the overall structure remains relatively dense. As indicated in Figure 3b(V), TiO_2_ particles incorporated into the outer layer are visible. Although some aggregation occurs, even in the aggregated state, the overall size of the particles remains in the range of several hundred nanometers. At the micron scale, TiO_2_ can be considered to be uniformly distributed within the TPU matrix. Figure 3b(VI) provides a closer look at the dispersed TiO_2_ particles, where the particle size is estimated to be ≈20–30 nm, taking into account the stacking of TiO_2_ and the effect of the gold sputtering process on the observation of nanoparticles.

Figure 3c illustrates the internal conductive layer of the TT/MT fiber. From Figure 3c(I),(II), it can be seen that the internal structure is highly porous, with a distinctive appearance that differs from normal foam structures. The interior is characterized by numerous interconnected voids in which the TPU‐MWCNT composite forms small stacked particles. As shown in Figure 3c(III), individual particles are roughly 5–10 µm in size, with irregular shapes, and the surface of each particle appears “hairy.” Upon further magnification, as depicted in Figure 3c(IV),(V), the surface is densely and uniformly coated with MWCNTs. This unique internal structure ensures that the fiber has not only an abundance of interconnected voids, but also a dense and uniform conductive network, contributing to its porous yet conductive properties.

In addition, for core‐shell structured fibers, strong adhesion between the core and the shell is crucial. Figure 3d effectively shows the core‐shell interface in the TT/MT fiber. Due to the same TPU material in both components, the core and shell exhibit excellent compatibility, to the point where a clear boundary between the two is indistinguishable. Only by observing the distribution of MWCNTs in the upper left corner and TiO_2_ nanoparticles in the lower right corner of the SEM image can the approximate location of the interface be inferred. This observation further demonstrates the perfect compatibility between the two components. Therefore, based on the SEM data of the fiber cross‐sections, it can be concluded that the TT/MT fibers have a thin, relatively dense outer layer, a porous and uniform conductive network inside, and excellent compatibility between the core and the shell, making them highly suitable as raw materials for fabricating adhesive double‐helical sensors.

**Figure 4:**
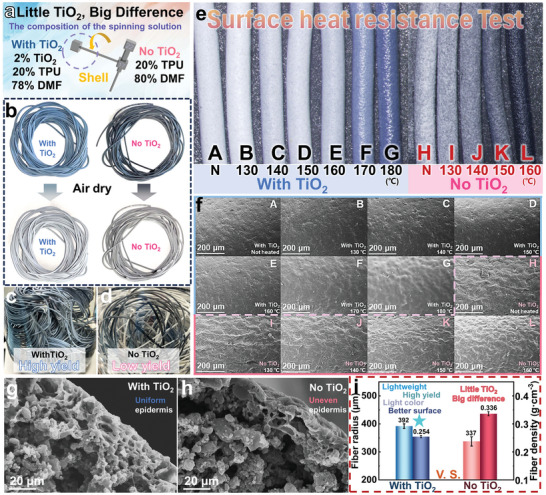
Importance of adding nano‐TiO_2_ in TT/MT fluffy core‐shell fibers: a) Little TiO_2_, big difference. b) Difference in fiber color after adding nano‐TiO_2_. c,d) The difference in spinnability of fibers after adding nano‐TiO_2_. e) Difference in the ability to withstand heat treatment of fibers after adding nano‐TiO_2_. f) Difference in epidermal microstructure after heating with nano‐TiO_2_ added. i) Difference in radius and density of fibers after adding nano‐TiO_2_.

**Figure** **4** provides a detailed illustration of the effects of incorporating nano‐TiO_2_ in our work. As indicated in Figure 4a, “Little TiO_2_, Big Difference”, under identical conditions except for the inclusion of nano‐TiO_2_ in the shell slurry, regular fibers (T/MT fibers) were prepared and compared to TT/MT fibers. The TT/MT fibers with TiO_2_ exhibit notable differences in color, preparation continuity, performance under heat‐treatment, surface structure, and especially in size and density compared to the regular fibers without TiO_2_.

As shown in Figure 4b, the TT/MT fibers are light blue‐black when wet and become white when drying, whereas the regular fibers are black when wet and gray when dry. The addition of TiO_2_ enhances the whiteness of the fibers. Furthermore, Figure 4c,d demonstrates that under the same coagulation bath, fibers with TiO_2_ can be produced continuously and stably, whereas those without TiO_2_ are discontinuous and unstable. This stability is attributed to the increased density of the spinning slurry with TiO_2_, which allows it to sink to the bottom of the container quickly for continuous spinning. In contrast, fibers without TiO_2_ have insufficient paste density, resulting in low production rates during spinning. In addition, during the process of forming a double‐helical structure by twisting and heat treating the fibers, it is necessary to heat the fiber surface to induce movement in the soft segments of the TPU. However, to ensure the integrity of the fiber structure, it is also essential that the surface structure and morphology of the fibers remain largely unaffected at this temperature. As depicted in Figure 4e, after 15 min of heat treatment at various temperatures, significant changes in color and morphology occur ≈170 °C for TT/MT fibers with TiO_2_, compared to ≈140 °C for regular fibers. To further investigate the surface structures of both fiber types under different heat treatments, SEM observations were conducted, as shown in Figure 4f (additional SEM images of the surface structures of TT/MT and regular fibers at various heat‐treatment temperatures are shown in Figures S4 and S5, Supporting Information). When subjected to higher temperature treatments, the fiber surfaces contract and become uneven. However, the inclusion of nano‐TiO_2_ allows the fiber surfaces to remain relatively smooth under heat treatment below ≈170 °C, making fiber less prone to damage under heat‐treatment. In addition, a TT/MT fiber and a T/MT fiber were twisted into a helical structure and subjected to identical heat treatment conditions. This approach ensured that both fibers experienced the same environment during the process. As shown in the optical microscope images (Figure S6, Supporting Information) and the cross‐sectional SEM images (Figure S7, Supporting Information), it is clearly observed that the T/MT fiber exhibits more significant shrinkage, deformation, and damage compared to the TT/MT fiber. These findings further corroborate the above conclusion that the incorporation of nano‐TiO_2_ enhances the physical performance of the TPU surface layer at elevated temperatures, making it less prone to shrinkage, deformation, and damage.

Moreover, the effect of nano‐TiO_2_ on the surface structure is evident from the SEM images of the fractured fiber cross‐sections (Figure 4g,h), which show smoother surfaces for the TT/MT fibers. Additionally, FESEM observations of the fiber surfaces under 1 kV accelerating voltage (as shown in Figure S8, Supporting Information) further confirm the smoother surface conclusion for fibers with nano‐TiO_2_. Most importantly, the inclusion of nano‐TiO_2_ changes the viscosity of the shell paste and the forming speed in the coagulation bath, thereby affecting the size and density of the fibers. As shown in Figure 4i, the inclusion of nano‐TiO_2_ increases the fiber (before heat treatment) radius by ≈16.3%, making the fibers fluffier and more compressible, with a reduced density of 0.254 g cm^−3^ compared to 0.336 g cm^−3^ for fibers without TiO_2_—a 25% decrease in density. Overall, the incorporation of nano‐TiO_2_ significantly affects various aspects of the fibers and improves the performance of the prepared TT/MT fibers in the fabrication of double‐helical sensors.

**Figure 5:**
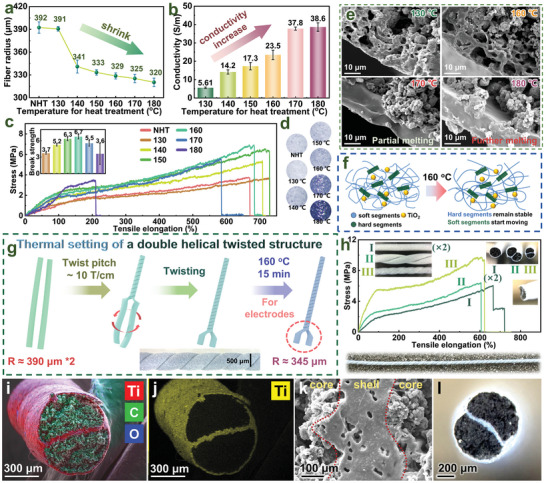
Exploration of preparation of double‐helical TT/MT fluffy core‐shell fibers: a) Degree of fiber shrinkage after heat treatment. b) Change in fiber conductivity after heat treatment. c) Change in mechanical strength of fibers after heat treatment. d) Color change of fibers after heat treatment. e) Sectional SEM of the external epidermis structure of fibers after heat treatment. f) Mechanism of fiber inner‐adhesion after heat treatment. g) Preparation of inner‐adhesive helical fibers. h) Comparison of tensile curves of inner‐adhesive helical fibers with ordinary fibers and partially helical fibers. i) Cross‐sectional view of the entire double‐helical fiber (colored according to EDS map). j) Ti element distribution in the cross‐section of the double‐helical fiber. k) SEM image of the binding site in the cross‐section of the double‐helical fiber. l) Microscopic image of the double‐helical fiber.

**Figure** **5** explores and analyzes the fabrication of a helical sensor with dual electrodes on the same side using TT/MT fibers. As depicted in Figure 5a, at temperatures above 130 °C, the TT/MT fibers experience some shrinkage, indicating increased movement of TPU molecular chains (NHT signifies No Heat Treatment). As the heat treatment temperature increases, the electrical conductivity of the fibers gradually improves (Figure 5b), driven by two key factors. First, the shrinkage of the fibers during heat treatment causes the internal conductive network to become more compact, enhancing conductivity. This effect is particularly evident in the conductivity improvements observed with heat treatments between 130 and 150 °C (The cross‐sectional SEM images of six fibers subjected to heat treatments at different temperatures are shown in the Figures S9–S13 (Supporting Information), with magnifications ranging from 100× to 10000×). Second, at higher temperatures, not only does the TPU on the fiber surface shrink, but the internal TPU within the fibers also contracts. This shrinkage reduces the specific surface area of the TPU, allowing the MWCNTs to better distribute across the surfaces of the TPU microparticles. (As observed in Figures S12 and S13 (Supporting Information), the relevant mechanisms are summarized in the schematic diagram presented in Figure S14, Supporting Information) This improved distribution enhances the conductivity of the fibers, as reflected in the conductivity gains achieved with heat treatments in the 150–170 °C range. Thermal treatment at different temperatures also affects the mechanical strength of the fibers, the stress‐strain curves in Figure 5c show an increase in strength due to heat treatment, although excessively high temperatures can damage the fibers (The thermogravimetric analysis of fibers is shown in Figure S1, Supporting Information 5). Furthermore, heat treatment changes the color of the fibers, as shown in Figure 5d. Further SEM observation of the cross‐sections of TT/MT fibers after heat treatment at different temperatures, as shown in Figure 5e, reveals that the skin structure of the fibers melts at 170 °C, which may cause damage. Considering the structure, strength, and conductivity of TT/MT fibers under various thermal conditions, a treatment temperature of 160 °C is chosen, as demonstrated in Figure 5f. At this temperature, nano‐TiO_2_ restricts the movement of the hard segments of TPU to some extent, while allowing the soft segments to move. When two TT/MT fibers are twisted, the internal stress at 160 °C is sufficient to enable thorough bonding of the TPU on the surfaces of the two fibers without causing damage. This process results in a strong internal bond within the double‐helical fiber.

Based on these findings, we designed a process for fabricating internally bonded double‐helical fibers, as shown in Figure 5g. Two fibers are twisted at a twist number of 10 T cm^−1^, and this structure is fixed by heat treatment at 160 °C for 15 min, resulting in an internally bonded double‐helical fiber structure. Notably, the cross section of the treated double‐helical fiber is approximately circular rather than the “8” shape typical of conventional double‐helical fibers, with a radius of ≈345 µm, which is even significantly smaller than that of a single TT/MT fiber before treatment due to the compressible nature of TT/MT fibers. Compared with traditional double‐helical structures, this nearly cylindrical structure not only has a smaller volume but also provides superior strength, which makes it suitable for weaving and knitting. As shown in Figure 5h, the tensile tests for two fibers treated at 160 °C (I), partially twisted fibers (with a twist level of ≈2 T cm^−1^) (II), and fibers prepared using the designed process (III) clearly demonstrate that the double‐helical fibers we designed exhibit higher stress strength. This improvement is primarily attributed to the significant reduction in their cross‐sectional area. To further observe and evaluate this double‐helical fiber structure, EDS was used for high‐resolution scanning, and SEM was color‐coded based on the elemental distribution map, resulting in Figure 5i, which visually shows the cross‐sectional structure of this double‐helical fiber. The distribution of Ti element is shown in Figure 5j, with more raw data in Figure S16 (Supporting Information). Evaluation of the bonding level at the contact areas of the two fibers in the SEM images of the fiber cross‐sections clearly shows complete and thorough bonding with a uniform distribution of nano‐TiO_2_ throughout the fused shell layer (Figure 5k), confirming the robustness and durability of this double‐helical fiber structure, with no risk of unraveling or internal short‐circuiting (more detailed SEM in Figure S17, Supporting Information). Microscopic observations in Figure 5l also reveal a well‐structured double‐helical fiber. Thus, this double‐helical fiber is highly suitable for use in flexible sensors with electrodes on the same side, which has a wide application potential.

**Figure 6:**
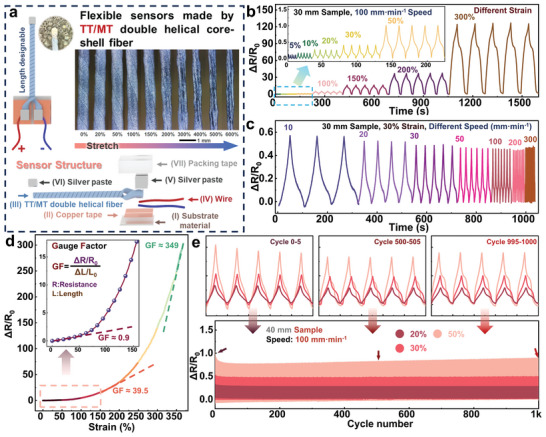
TT/MT double‐helical fiber stretch sensing performance: a) Schematic of stretch sensor made from TT/MT double‐helical fibers. b) Stretch sensing performance of TT/MT double‐helical fibers under different strain amplitudes. c) Stretch sensing performance of TT/MT double‐helical fibers under different stretching speeds. d) Stretch sensing curve of TT/MT double‐helical fibers. e) 1000 cycle stretch sensing curve of TT/MT double‐helical fibers under different strain amplitudes.

**Figure** **6** illustrates the performance of the double‐helical flexible sensor with two electrodes on the same side. Figure 6a shows the structure of this flexible sensor. Two conductive copper tapes (II) are attached to a substrate (I), and one end of the double‐helical fiber (III), prepared as described in Figure 5g, together with two wires (IV), is fixed to the copper tapes using conductive silver paste (V). The other end is also bonded with conductive silver paste (VI). After curing the conductive silver paste in an oven at 100 °C for 30 min, packing tape is applied to the electrodes to protect them. When the flexible sensor is operating, current flows from the positive electrode into one strand of the double‐helical fiber travels to the other end through the connection created by the conductive silver paste, and then flows into the second strand of the double‐helical fiber before exiting through the negative electrode. Any deformation in the fiber causes changes in the internal conductive network, resulting in a change in resistance, thus allowing for flexible strain sensing. Furthermore, the double‐helical structure does not unwind under strain conditions from 0% to 600% due to its strong and stable internal adhesion. (See Movie S2 and Figure S18 (Supporting Information) for related videos and images).

As shown in Figure 6b, this flexible sensor operates stably under different strain ranges from 0% to 300%, and the resistance change ratio (ΔR/R_0_) increases consistently with the applied strain. Under different strain speeds, the sensor maintains good performance, as illustrated in Figure 6c. At the same strain of 30%, the resistance change ratio remains relatively stable at different stretching speeds ranging from 10 to 300 mm min^−1^, indicating that the sensor works reliably at different speeds. The strain‐resistance change rate curve of this flexible sensor made from TT/MT fibers is presented in Figure 6d, which shows an approximately exponential trend. This curve can be divided into three sections to calculate the Gauge Factor (GF). In the small strain range (within 50% strain), the GF is ≈0.9. In the big strain range (≈150% strain), the GF is ≈39.5. At very large strains (> 300% strain), the GF reaches ≈349. Additionally, as joint movements during human motion detection typically cause 20–50% strain on flexible sensors, cyclic tensile tests were conducted on this flexible sensor made of TT/MT fibers. As shown in Figure 6e, the sensor exhibits stable and reliable response signals within 1000 cycles, demonstrating its long‐term operability and durability. In addition, detailed hysteresis information and the corresponding mechanism analysis will be presented in Figure S19 (Supporting Information). As shown in Figure S20 (Supporting Information), considering the mechanical movement time of ≈60 ms, the total response times for TT/MT double‐helical fibers under stretching and recovery are ≈102 and 105 ms, respectively. The TT/MT double‐helical fiber sensor demonstrated good performance under varying humidity and temperature conditions, as well as a certain degree of washability. The corresponding tests are presented in Figures S21–S23 (Supporting Information). Based on the data in Figure 6, it can be concluded that the double‐helical flexible sensor made of TT/MT fibers exhibits excellent performance.

**Figure 7:**
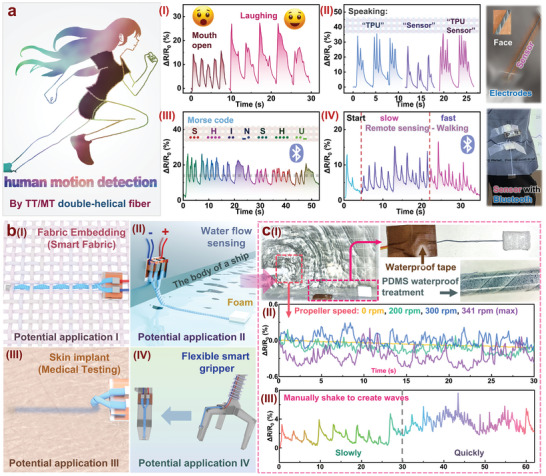
Actual and potential application of double‐helical TT/MT fibrous sensor: a) Human motion sensing, including facial motion recognition (i), voice recognition (ii), Morse code recognition (iii), and motion recognition (iv). b) Potential applications, including fabric embedding (i), water flow sensing (ii), skin implant (iii), and flexible smart gripper (iv). c) An example of the specific implementation of water flow sensing.

The double‐helical sensor, made of TT/MT fibers with two electrodes on the same side, enables a variety of meaningful applications. First, as illustrated in **Figure** **7**a, this flexible sensor can be used to monitor human motion. When attached to a person's cheek, it can detect mouth movements. As shown in Figure 7a(I),(II), the sensor can track the wearer's mouth opening or laughing as well as facial muscle activity by measuring changes in resistance. This functionality can be applied in emotion recognition and sleep breathing tests. In addition, the signal waveform of the sensor can identify speech characteristics, which is beneficial for speech recognition in non‐verbal individuals. Additionally, the TT/MT double‐helical fiber can achieve remote sensing functionality when integrated with a microcontroller featuring built‐in Bluetooth capabilities. A photograph of the setup is provided in Figure S24 (Supporting Information). As a demonstration, in Figure 7a(iii) and Movie S3 (Supporting Information), we show the fiber being pulled by hand and held for varying durations (short or long). The corresponding signals are displayed in real‐time on computer software, enabling the remote wireless transmission of Morse code. Furthermore, as illustrated in Figure 7a(iv), the system was applied to the human knee joint, with the device attached to the surface of the pants. This setup allowed for real‐time remote monitoring of the wearer's gait movements. To better showcase this functionality, we used a projector and recorded a related video, provided as Movie S4 (Supporting Information).

To further highlight the unique capabilities of this flexible sensor, Figure 7b outlines four potential applications: (I) integration into fabrics to create smart textiles, where electrical connections are needed at only one location. The photo demonstration of TT/MT double‐helical fibers embedded in knitted and woven fabrics is shown in Figure S25 (Supporting Information). In the future, elastic fibers with modulus and properties matching those of the TT/MT double‐helical fibers can be designed, paving the way for the development of smart fabrics; (II) water flow sensing, which may be difficult for conventional sensors to realize;(III) Subcutaneous signal detection: Compared to traditional flexible sensors with larger volumes, our TT/MT double‐helical fiber offers distinct advantages, including a small diameter and the absence of electrodes on one side. This unique design has the potential to be implanted using interventional catheters rather than requiring surgical procedures, thereby minimizing harm to the human body. Such features provide valuable insights and structural design solutions for future research in subcutaneous sensing applications; and (IV) monitoring the motion of soft robotic grippers without the need for circuit connections at the tips of the grippers, which would otherwise reduce their sensitivity.

To demonstrate the practical feasibility of these potential applications, we selected water flow detection for further implementation and testing. Figure 7c(I) illustrates a schematic of the water flow detection setup. After a simple waterproofing treatment, the TT/MT double‐helical flexible sensor can be used for real‐time water flow monitoring. (14‐day salt water and pure water immersion experiment was shown in Figure S26, Supporting Information) Figure 7c(II) shows the sensing signals obtained by the flexible sensor under varying degrees of water agitation caused by a propeller. When the propeller is off, the resistance of the TT/MT double‐helical fiber remains stable as a nearly flat line. However, as the propeller operates at different speeds, the water waves induce vibrations in the TT/MT fiber (as demonstrated in Movie S5, Supporting Information), producing distinct signals. Additionally, as shown in Figure 7c(III), when larger waves are manually generated by stirring the water surface, the TT/MT double‐helical fiber accurately captures real‐time signals (as shown in Movie S6, Supporting Information). This application holds significant promise for future water surface monitoring technologies. In summary, Figure 7 demonstrates that flexible sensors made from TT/MT fibers not only enhance the functions of traditional flexible sensors, but also enable a range of novel applications due to their free‐ended design.

**Figure 8:**
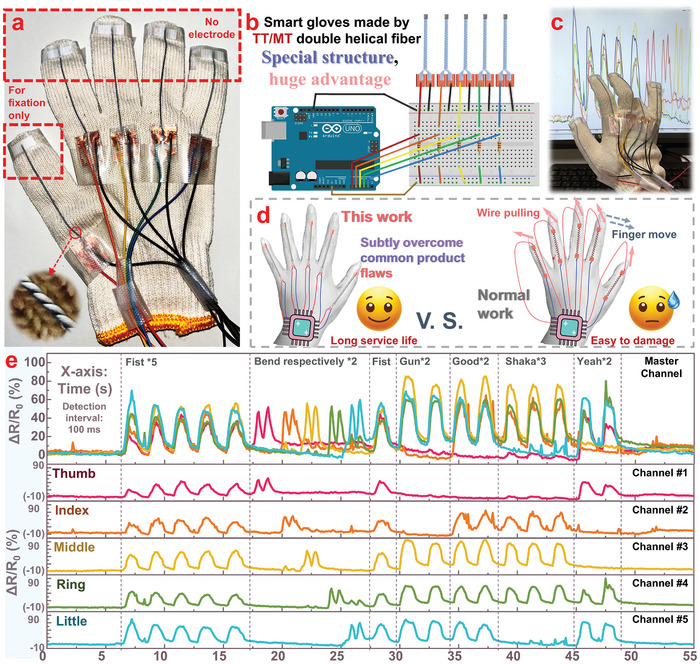
Demonstration of TT/MT double‐helical fibers applied in the preparation of smart gloves: a) Photo of the smart glove. b) Circuit diagram of the smart glove. c) Photo during testing. d) Display of the advantages of double‐helical fiber structure sensors. e) Signals of different gestures.

**Figure 9:**
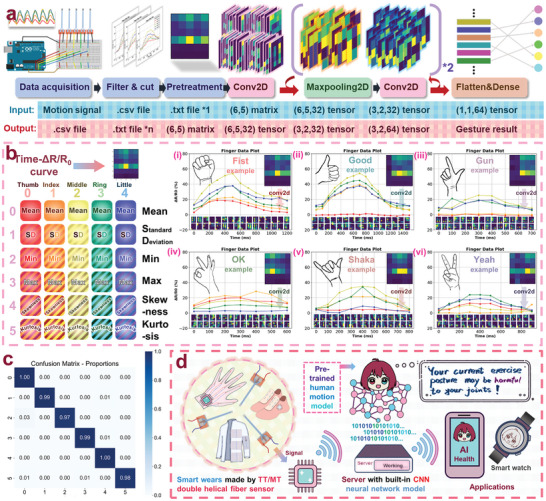
Gesture recognition in smart gloves fabricated using TT/MT double‐helical fiber sensors with CNN machine learning:a) Flowchart. b) Methods of data preprocessing and examples of different gesture signals. c) Gesture recognition accuracy rate (confusion matrix). d) Future application scenarios of this research.

Currently, smart gloves with gesture recognition functionality are attracting increasing attention from researchers in the field of flexible sensors due to their significant practical value. However, the complexity of fabricating multi‐channel smart gloves and processing their signals has caused many researchers to opt for simpler recognition applications using only single‐channel sensors,^[^ ^39^, ^40^ ^]^ and only a few of researchers^[^ ^41^, ^42^ ^]^ use multi‐channel smart gloves for recognition applications. With the advantages of the double‐helical flexible sensor made of TT/MT fibers, which features two electrodes on the same side for easy fabrication and wiring, we can easily design multi‐channel smart gloves and implement complex gesture recognition applications based on CNN machine learning. The relevant contents are shown in **Figures** **8**, **9**.

Figure 8a shows a photo of the smart glove we designed. Each of the five fingers is equipped with a flexible sensor made of TT/MT fibers. Contrary to common wiring methods for flexible sensors, we connected both the positive and negative electrodes on the back of the hand. The positive electrodes for the five fingers are wired with color‐coded wires: red, orange, yellow, green, and blue, while the negative electrodes all share a black wire. On the fingers, the fibers are attached only to the fingertips. During hand movements, the fingers are in constant motion, while the back of the hand remains relatively still. The key advantage of this design is that it minimizes the pulling and potential damage to the electrodes caused by movement or shaking, significantly increasing the comfort and durability of the smart glove. A photo of the entire smart glove device is shown in Figure S27 (Supporting Information).

We used an Arduino microcontroller to convert the five channels of electrical signals from the glove into digital signals for the computer (the circuit wiring diagram is shown in Figure 8b). These signals are then displayed on the computer through a user interface developed using Python PyQt5 (as shown in Figure 8c). Figure 8d uses a schematic diagram to show how our innovative method addresses common challenges in gesture recognition applications of flexible sensors. We utilized an Arduino microcontroller to control the robotic hand, enabling the hand to open and close its palm once per second (the corresponding code can be found in Figure S28, Supporting Information). Using this method, we conducted ≈2500 cycles of testing on TT/MT double‐helical fibers and ordinary fibers (TT/MT fibers without twist treatment or heat treatment), further demonstrating the superior performance of TT/MT double‐helical fibers compared to ordinary fibers, See Figure S29 (Supporting Information) for detailed information. As shown in Figure 8e, we tested the smart glove design, with related video footage provided in Movie S7 (Supporting Information). When different gestures are made, the five channels respond differently depending on the movement of each finger, and for the same gesture, the signal is highly repeatable. These test results indicate that the flexible sensor made of TT/MT fibers can be effectively used in the development of complex smart devices such as smart gloves, demonstrating excellent performance in practical applications.

To further demonstrate the practical application value of TT/MT fibers, we present a “gesture recognition” function and future prospects in Figure 9. Since the smart glove designed using TT/MT fibers can reliably output five channels of signals, we employed CNN for machine learning to analyze these signals. As shown in Figure 9a, after gesture signals are converted into digital data by an Arduino microcontroller, the data are segmented by a program to transform the continuous five‐channel (Time‐ΔR/R_0_) curves containing multiple gestures into individual 2D arrays, each of which contains all data within the time range of a single gesture, to be used as training or testing data. In addition, since CNN requires the application of convolutional kernels on a regular grid, and the duration of gestures varies depending on the speed of each movement, we need to preprocess the gesture data. Therefore, we processed the data into a 5 × 6 matrix and applied several convolution and pooling steps. From these steps, the data features are extracted to classify the initial gesture movement, achieving the functionality where “after wearing the smart glove, the computer can automatically recognize your gestures” (detail of CNN machine learning process is shown in Figure S30, Supporting Information).

As shown in Figure 9b, we explain the data preprocessing method in detail. The smart glove contains five sensors representing five fingers, but the time taken to perform a gesture can vary from 0.5 to 2 s. The data are collected at 100 ms intervals, so the initial data structure is a 5 × n (5 < n< 20) 2D array. To standardize these data, we extract six core features—mean, standard deviation, minimum, maximum, skewness, and kurtosis—from the ΔR/R_0_ for each finger over time, thus normalizing the data while preserving their essential features. In addition, we illustrate the gesture data of six common gestures (shown in Figure S31, Supporting Information) and their preprocessed matrices on the right side of Figure 9b for intuitive display. Currently, we have collected 1200 gesture data samples as a dataset, which includes 200 samples for each of the six common gestures. We used 50% of the dataset as a training set and 50% as a test set to conduct gesture recognition application tests based on CNN machine learning. As shown in Figure 9c, the recognition accuracy of the model trained on the training set reached 98.8% when tested on the test set (593 correct identifications out of 600 test samples). This demonstrates that the smart glove, equipped with flexible sensors made from TT/MT fibers, can achieve high accuracy gesture recognition through CNN machine learning algorithms.

As shown in Figure 9d, flexible sensors made of TT/MT fibers have significant future potential, especially as computer hardware, machine learning algorithms, and Internet technologies evolve. For instance, smart gloves, shoes, and clothing embedded with flexible sensors made of TT/MT fibers can convert physical signals from different parts of the body into electrical signals, and transmit them to a server via Bluetooth or remote data devices. The server, using pre‐trained CNN models, can convert these complex signals into suggestions about personal health status, posture, and more. These suggestions can then be sent to an individual's smartphone or smartwatch, improving the quality of daily life through flexible sensor technology.

## 4 Conclusion

This work presents a novel approach to the design and fabrication of flexible sensors with significant potential for various applications in the field. We present a comparative table in Table S1 (Supporting Information), summarizing relevant studies from the past year.^[^ ^16^, ^34^, ^43^, ^44^, ^45^, ^46^ ^]^ Compared to other fiber‐shaped electronic devices published in 2024, our work achieves comparable or slightly superior sensor sensitivity. Additionally, through the innovative design of a same‐side dual‐electrode double‐helical structure, we address the critical issue of electrode damage in flexible sensors caused by joint movement. Furthermore, compared to other double‐helical flexible sensors, the diameter of our fiber has been greatly reduced, while the conductive material volume fraction has been increased to ≈90%. Three key innovations are highlighted in this study: (1) The unique design of an internally encapsulated double‐helical structure. Inspired by the hydrogen bonds in DNA, the shell structure with TPU softens and adheres under certain conditions, creating a durable double‐helical structure that remains intact, making it ideal for the fabrication of single‐sided, dual‐electrode fiber sensors, provides a new idea for highly integrated smart fabrics in the future. (2) The introduction of nano titanium dioxide (TiO_2_) into the coaxial wet‐spinning process. By adjusting the density and viscosity of the slurry, we obtained fibers with a fluffy and porous structure, which are more resistant to heat treatment and have higher whiteness, making them well suited for the construction of internally encapsulated double‐helical TT/MT fibers. (3) The design of an intelligent glove and the customization of a CNN machine learning model. By integrating multidisciplinary approaches, we have successfully applied the TT/MT fibers to gesture recognition with high accuracy, providing a promising route to the commercialization and practical application of flexible sensors.

However, there is still room for improvement due to certain practical limitations. For example, the production efficiency of wet‐spinning in the laboratory remains slow. Future efforts could focus on scaling up the production of TT/MT fibers, possibly using customized equipment to improve manufacturing efficiency. In addition, processes such as heat treatment, and sensor fabrication are currently manual. Automating these steps through mechanical equipment could significantly reduce labor costs, and unlock the full commercial potential of double‐helical TT/MT fiber‐based flexible sensors.

## Conflict of Interest

The authors declare no conflict of interest.

## Supporting information



Supporting Information

Supplemental Movie 1

Supplemental Movie 2

Supplemental Movie 3

Supplemental Movie 4

Supplemental Movie 5

Supplemental Movie 6

Supplemental Movie 7

## Data Availability

The data that support the findings of this study are available from the corresponding author upon reasonable request.
