# Supplementary material for: Structure and Wiring Optimized TT/MT Double‐Helical Fiber Sensors: Fabrication and Applications in Human Motion Monitoring and Gesture Recognition
Source: Adv Sci (Weinh). 2025 Feb 4;12(12):2416564. doi: 10.1002/advs.202416564 (PMC11948013; doi:10.1002/advs.202416564)
Supplement: Supplementary file 1 — Supporting Information [file ADVS-12-2416564-s006.docx]

**Structure and Wiring Optimized TT/MT Double-Helical Fiber Sensors:** **Fabrication and Applications in Human Motion Monitoring and Gesture Recognition**

Ziwei Chen^a,b^, Daoxiong Qian^b^, Dandan Xie^a,b^, Chunxia Gao^c^, Jian Shi^a,b,d^, Hideaki Morikawa^a,d^, Chunhong Zhu^a,b,d,*^

^a^ Graduate School of Medicine, Science and Technology, Shinshu University, Tokida, Ueda, Nagano 386-8567, Japan

^b^ Institute for Fiber Engineering and Science (IFES), Shinshu University, 3-15-1 Tokida, Ueda, Nagano 386-8567, Japan

^c^ School of Chemistry and Chemical Engineering, Yangzhou University, Yangzhou 225002, China

^d^ Faculty of Textile Science and Technology, Shinshu University, 3-15-1 Tokida, Ueda, Nagano 386-8567, Japan

* Corresponding author at: Institute for Fiber Engineering and Science (IFES), Shinshu University, Tokida, Ueda, Nagano 386-8567, Japan.

E-mail address: zhu@shinshu-u.ac.jp (C. Zhu).


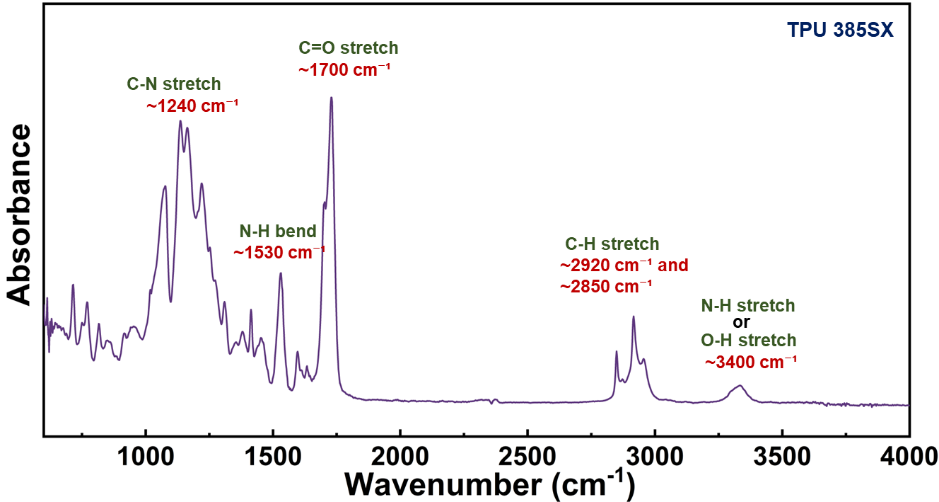


**Figure S1**: The infrared spectrum of raw material TPU.

**~3400 cm⁻¹ Peak:** The broad peak around ~3400 cm⁻¹ typically indicates N-H stretching vibrations, characteristic of urethane linkages in TPU. Additionally, this peak may include contributions from O-H stretching vibrations, often due to moisture or the presence of hydroxyl groups within the material.

**~1700 cm⁻¹ Peak:** A sharp peak in this region is characteristic of C=O stretching vibrations, confirming the presence of carbonyl groups in the urethane linkages of polyurethane.

**~2920 cm⁻¹ and ~2850 cm⁻¹ Peaks:** These peaks correspond to asymmetric and symmetric C-H stretching vibrations, respectively, and are commonly associated with methylene groups (-CH₂-) in the polyurethane backbone.

**~1530 cm⁻¹ Peak:** This peak may be attributed to N-H bending vibrations, indicative of urethane linkages within the polymer structure.

**~1240 cm⁻¹ Peak:** This peak corresponds to C-N stretching vibrations, further confirming the presence of urethane (carbamate) linkages in the polyurethane.


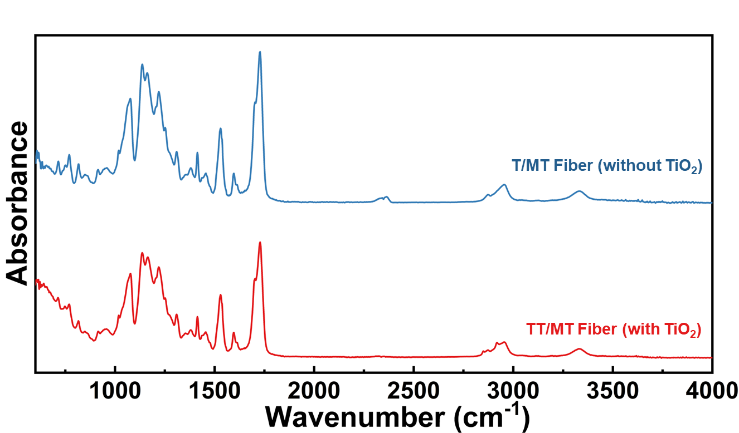


**Figure S2:** The infrared spectrum of TT/MT fiber and regular T/MT fiber (without TiO_2_)

After the addition of nano TiO_2_, the enhanced infrared absorption of TT/MT fibers below 600 cm⁻¹ is likely caused by lattice vibrations or low-frequency vibrations of Ti–O bonds induced by the nanoparticles.


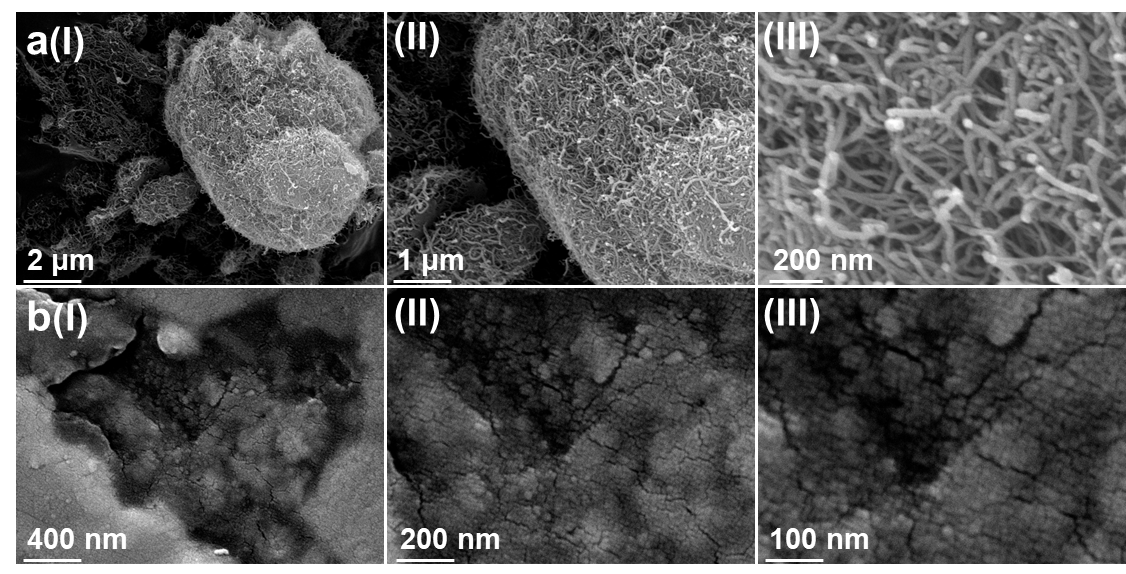


**Figure S3:** The SEM image of the material used in this paper: (a) MWCNT, (b)TiO_2_

These materials were subjected to gold sputtering treatment for 60 seconds before being observed using FESEM.


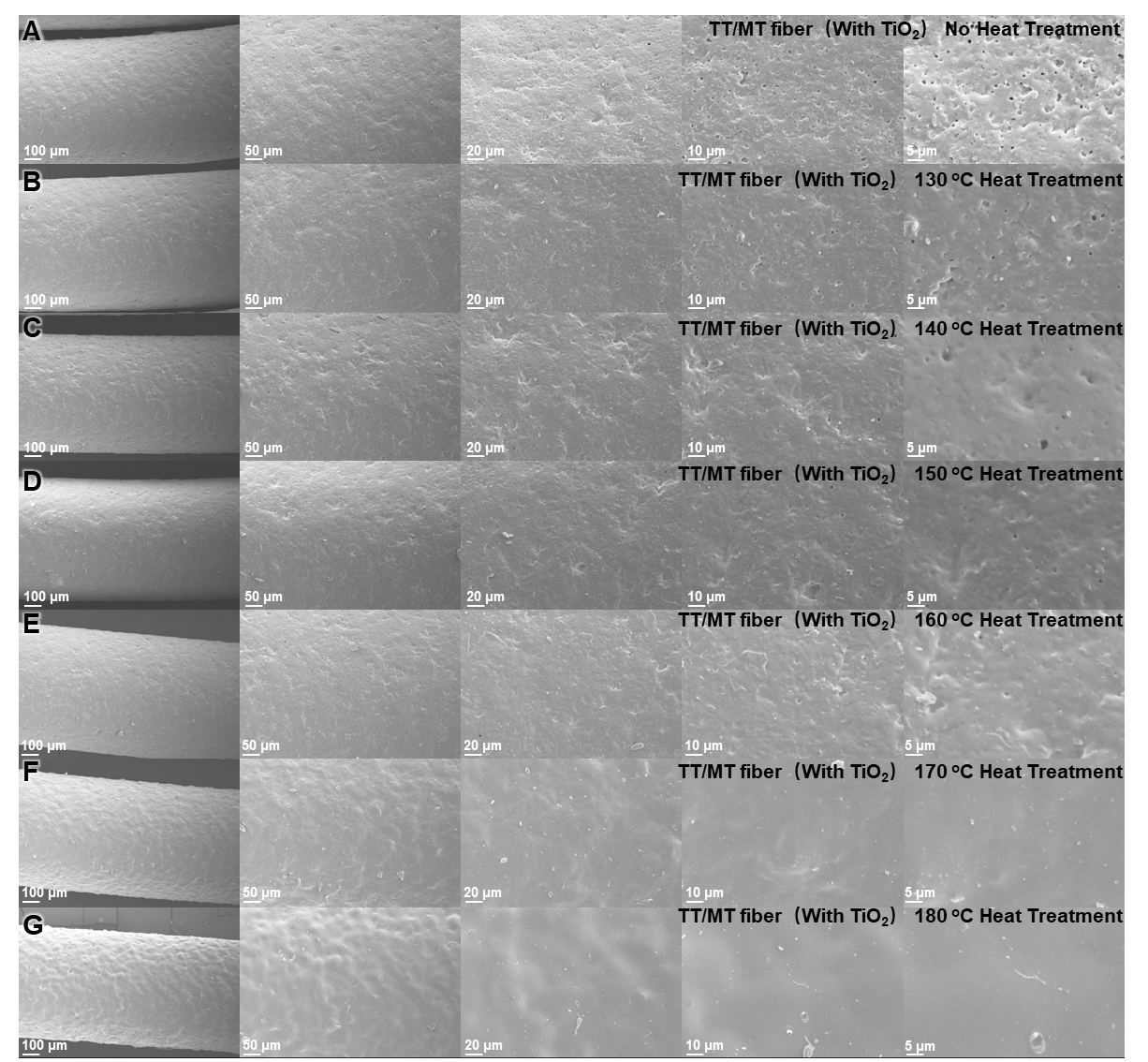


**Figure S4**: The detailed SEM image of the surface of TT/MT fibers under different heat-treatment temperature: (a) no heat-treatment, (b) 130 ^o^C, (c) 140 ^o^C, (d) 150 ^o^C, (e) 160 ^o^C, (f) 170 ^o^C, (g)180 ^o^C.

For TT/MT fibers containing nano TiO_2_, as shown in **Fig.S4** it can be observed that surface structural changes occur at 170°C and above. Specifically, the fibers become thinner, exhibit increased roughness at a scale of 100 μm, and lose their surface pore microstructure at a scale of 10 μm. This may be due to the melting of the TPU component caused by the elevated temperature.


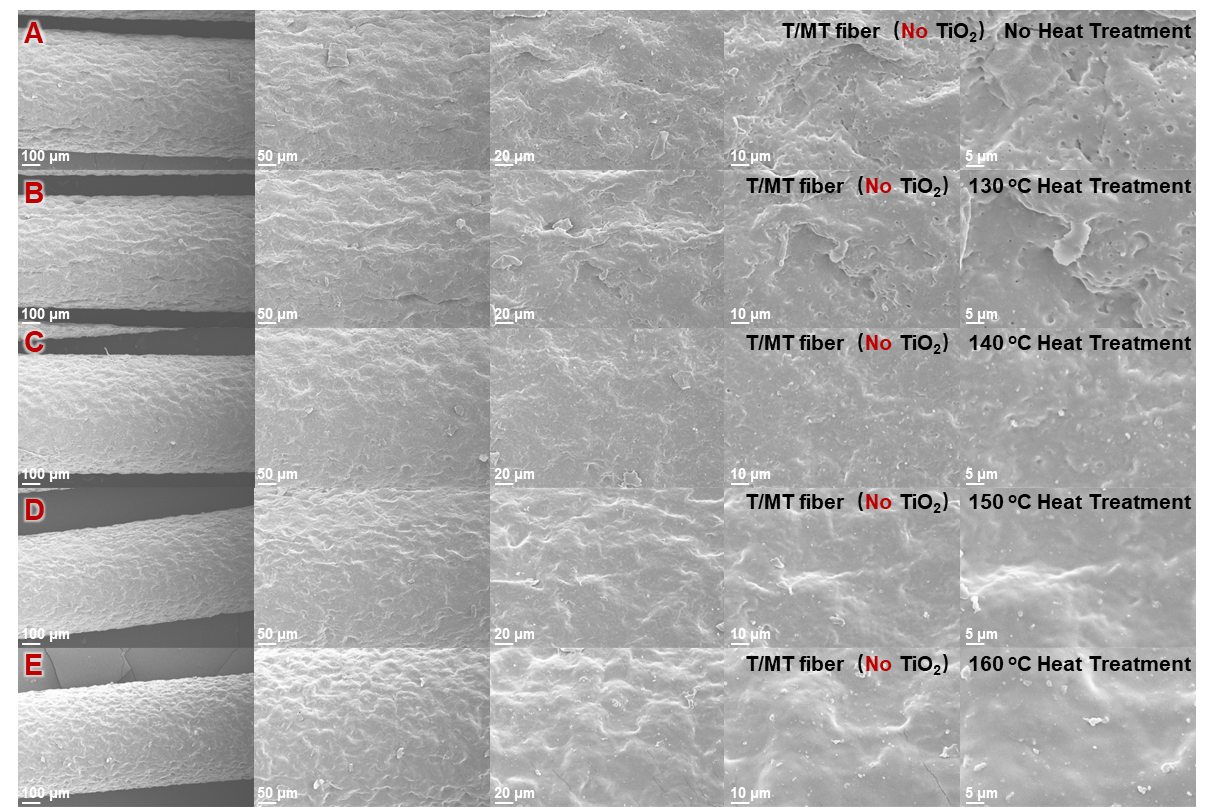


**Figure S5:** The detailed SEM image of the surface of T/MT fibers under different heat-treatment temperature: (a) no heat-treatment, (b) 130 ^o^C, (c) 140 ^o^C, (d) 150 ^o^C, (e) 160 ^o^C.

For regular T/MT fibers without nano TiO_2_, as shown in **Fig.S5**, compared to **Fig.S4**, it can be observed that the fibers themselves are relatively thin and have a wrinkled surface structure. When the temperature reaches 150 °C or higher, the fibers visibly become thinner, and the surface becomes noticeably rougher. On a 10 μm scale, the surface structure shows significant changes compared to samples that have not undergone heat treatment.


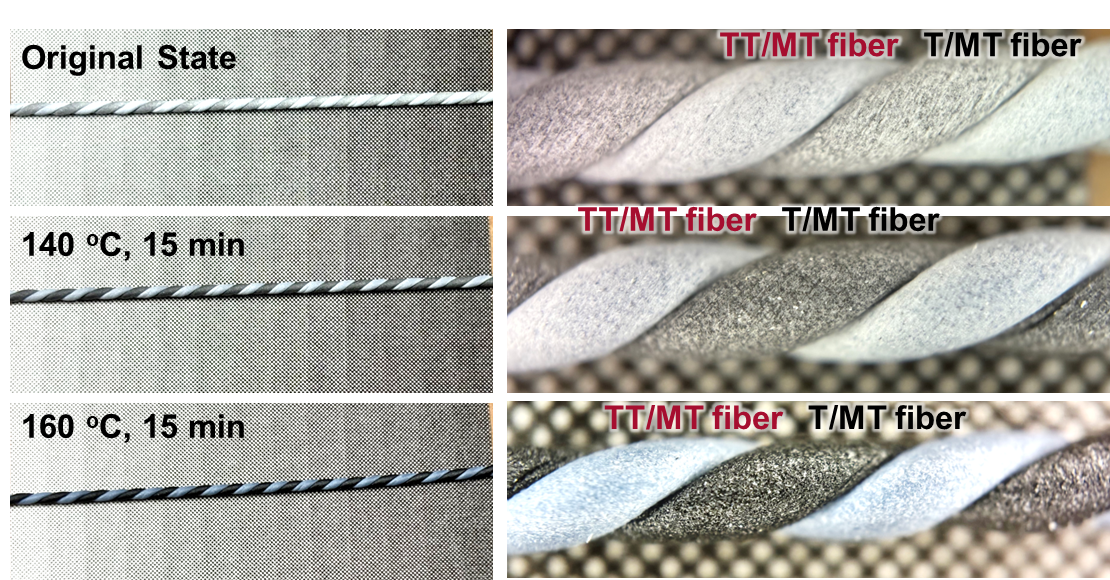


**Figure.S6** A double-helical fiber composed of a T/MT fiber and a TT/MT fiber was used for characterization to visually and intuitively demonstrate the significance of the addition of titanium dioxide.

As shown in **Fig.S6**, a double-helical fiber composed of a T/MT fiber and a TT/MT fiber was used to observe morphological changes under initial conditions and after heat treatment at different temperatures. At 140 °C, the T/MT fiber surface exhibited noticeable discoloration, indicating alterations in its physical structure. At 160 °C, the TT/MT fiber surface showed only a slight blue tint, whereas the T/MT fiber surface turned black and glossy, suggesting a complete structural change that could result in damage or potential short circuits. Heat treatment at 160 °C does not damage the TT/MT fiber; instead, it activates the soft segments in its surface layer, allowing it to bond effectively with other fibers without compromising its integrity.


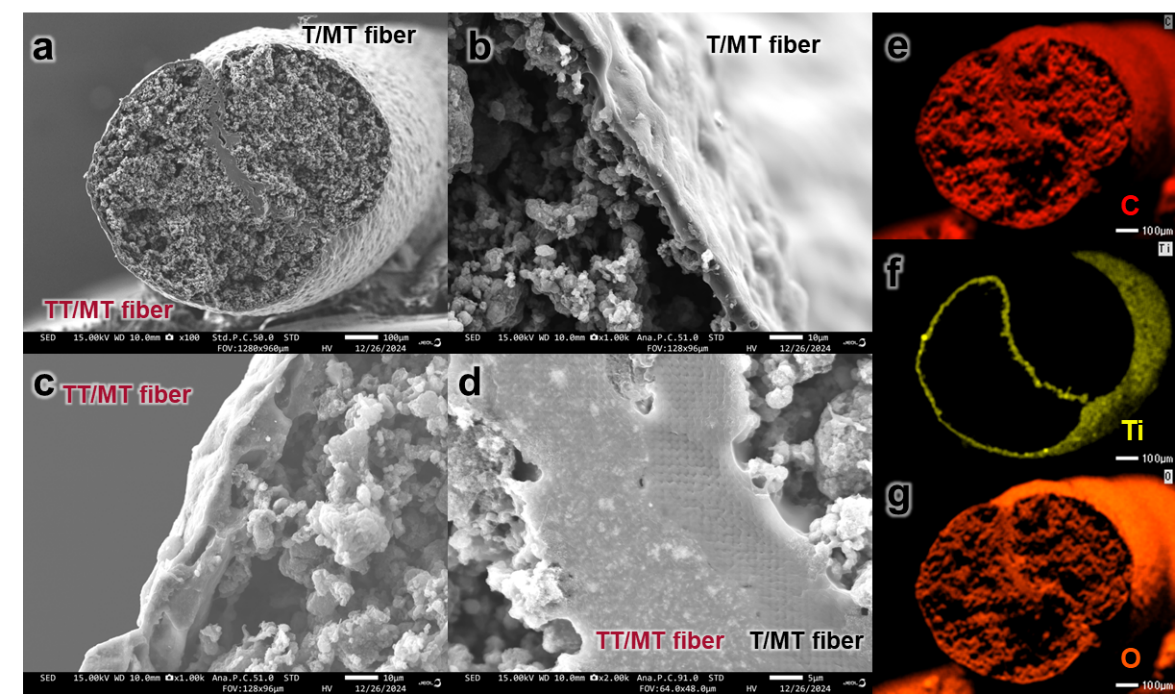


**Figure.S7** Cross-sectional SEM images of a double-helical fiber comprising a T/MT fiber and a TT/MT fiber prepared via heat treatment at 160 °C. (a) Overview of the fiber cross-section, (b) SEM image of the epidermis on the T/MT fiber side, (c) SEM image of the epidermis on the TT/MT fiber side, (d) SEM image of the interface where the two fibers merge internally, and (e-g) EDS mapping of the double-helical fiber.

As shown in **Fig.S7(a)**, the cross-sectional SEM image depicts a double-helical fiber composed of a T/MT fiber and a TT/MT fiber, prepared via heat treatment at 160 °C. The thicker region in the lower left corresponds to the TT/MT fiber, which contains TiO_2_, while the thinner region in the upper right corresponds to the T/MT fiber, which does not contain TiO_2_. It can be observed that under heat treatment at 160 °C, the epidermis of the T/MT fiber experienced greater shrinkage compared to the TT/MT fiber.

Furthermore, by comparing **Fig.S7(b)** and **Fig.S7(c)**, it is evident that the surface of the T/MT fiber is significantly uneven and shows signs of damage, further confirming the effect of nano-TiO_2_ incorporation. In **Fig.S7(d)**, TiO_2_ particles can be observed in the cross-section of the TT/MT fiber at the lower right. These particles restrict the large-scale movement of TPU molecules at elevated temperatures, thereby enhancing the physical performance of TPU under such conditions. **Figs.S7(e-g)** present EDS mapping, clearly illustrating the distribution of Ti elements. The SEM images provide further visual evidence that the incorporation of nano-TiO_2_ enhances the physical performance of the TPU surface layer at elevated temperatures, making it less prone to damage.


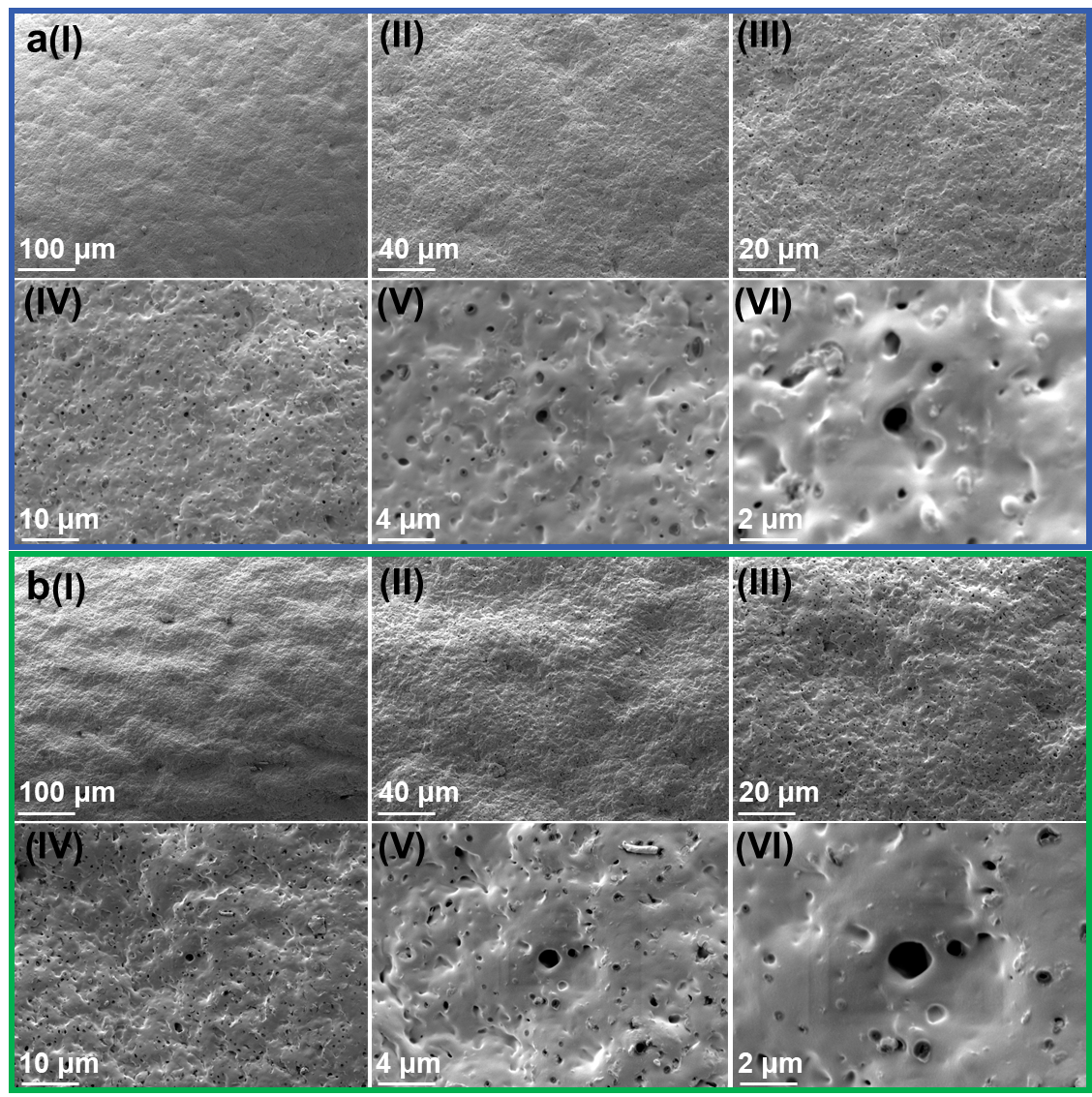


**Figure S8:** The detailed FESEM image of the surface of TT/MT fibers and regular T/MT fibers (no TiO_2_) under 1 kV accelerating voltage: (a) TT/MT fibers, (b) Regular T/MT fibers.

Further observations in **Fig.S8** suggest that the addition of TiO_2_ significantly smoothens the surface of the fibers, and at the microscopic level, the size of the micropores on the fiber surface becomes more uniform. This may be due to the addition of TiO_2_, which increases the system's viscosity, and stabilizes the TPU polymer chains. During the wet spinning process, the higher viscosity and more stabilized polymer chains result in a more intact and smoother fiber shape, reducing the likelihood of shrinkage.


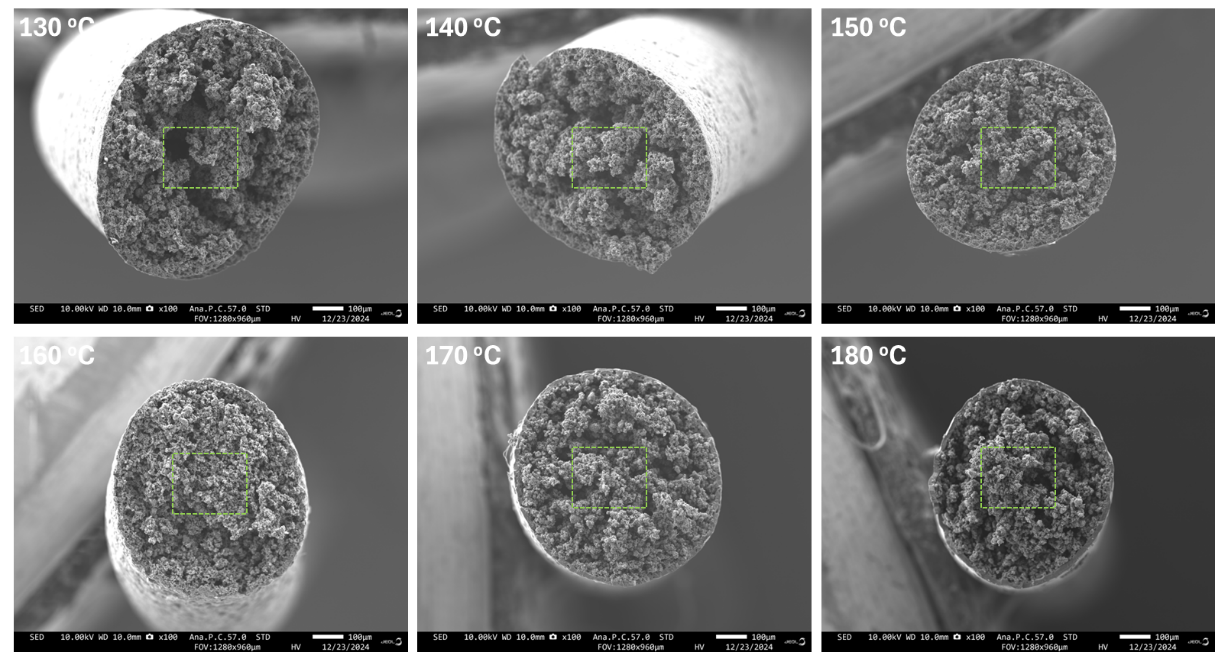


**Figure S9:** The cross-sectional FESEM images of the six fibers subjected to different heat treatment temperatures (130 °C to 180 °C), at a magnification of 100×.

As shown in **Fig.S9** , this is an SEM image of the fibers at 100× magnification. It is evident that with increasing heat treatment temperature, the fibers exhibit a trend of shrinkage (a gradual reduction in diameter). Additionally, the internal MWCNT/TPU fluffy structures within the fibers are observed to compress and become more compact as the heat treatment temperature rises.

For instance, in samples treated at 130°C, small cracks can still be observed in the center of the fibers (these cracks are also visible in **Fig.3a** of the main text), which may contribute to reduced conductivity. This compression and compaction can, to some extent, enhance the fiber's conductivity. This phenomenon likely explains the decrease in fiber resistance with heat treatment. Specifically, this is a key reason why the electrical conductivity of the fibers increases from approximately 5.61 S/m at 130°C to 17.3 S/m at 150°C.


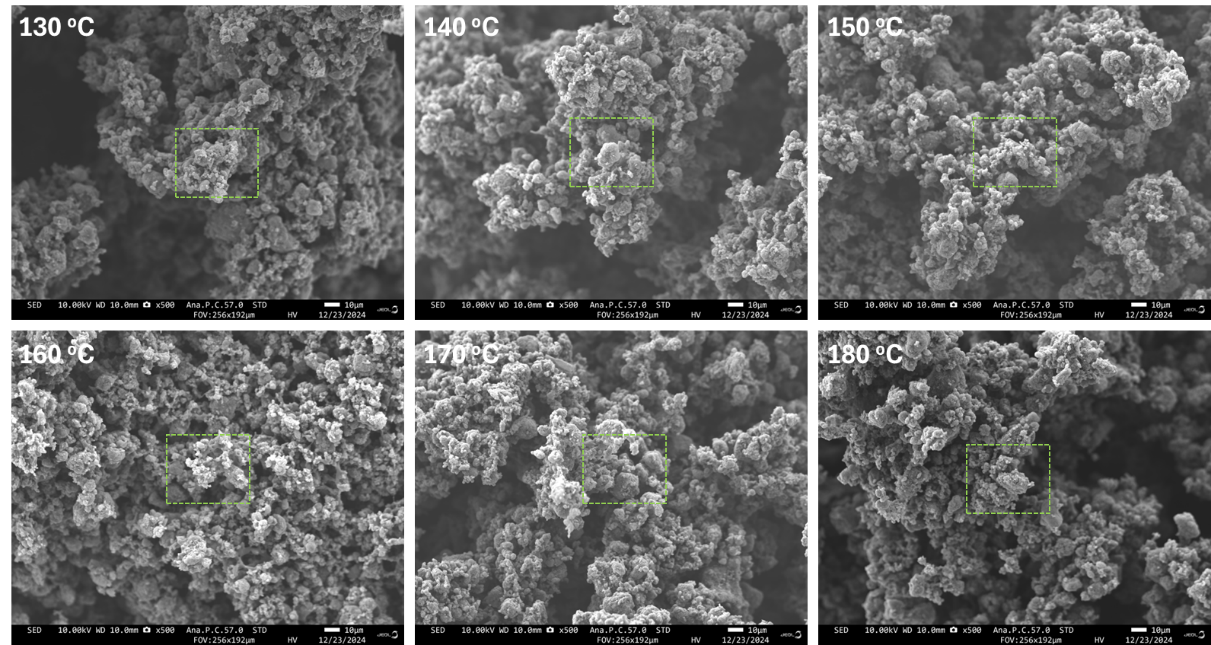


**Figure S10:** The cross-sectional FESEM images of the six fibers subjected to different heat treatment temperatures (130 °C to 180 °C), at a magnification of 500×.

The SEM images of the six fibers were further magnified in the areas highlighted by green dashed boxes (from **Fig.S9** to **Fig.S11**). As shown in the **Fig.S10** SEM micrographs were obtained at 500×


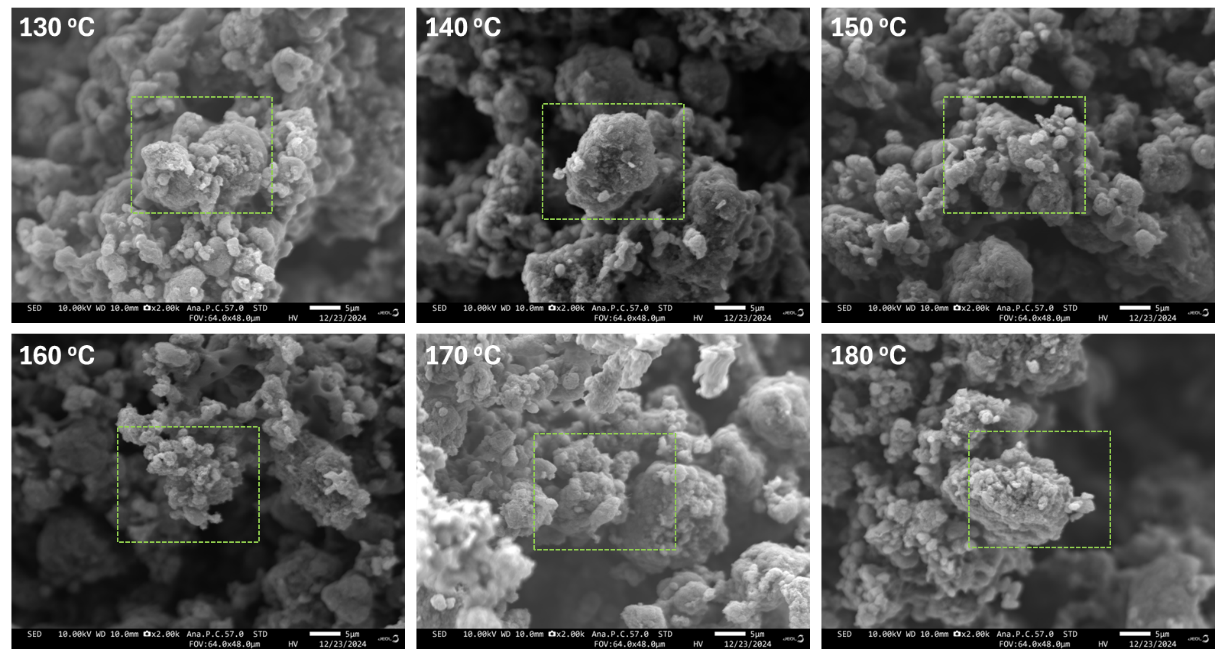


**Figure S11:** The cross-sectional FESEM images of the six fibers subjected to different heat treatment temperatures (130 °C to 180 °C), at a magnification of 2000×.


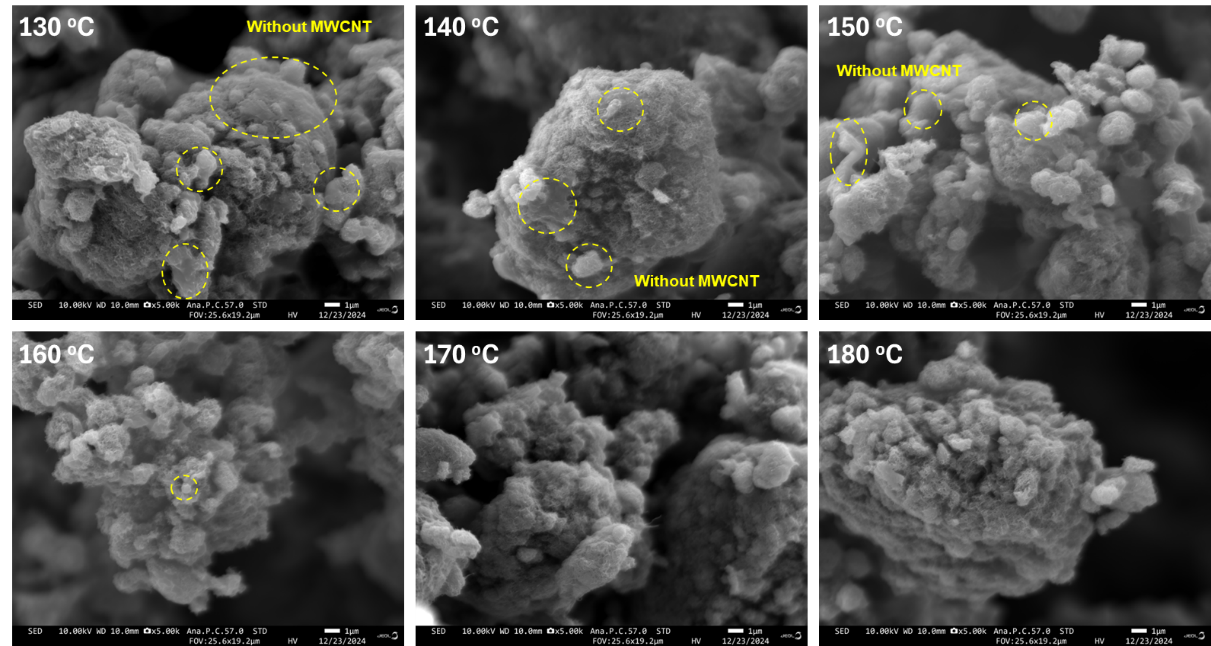


**Figure S12:** The cross-sectional FESEM images of the six fibers subjected to different heat treatment temperatures (130 °C to 180 °C), at a magnification of 5000×.


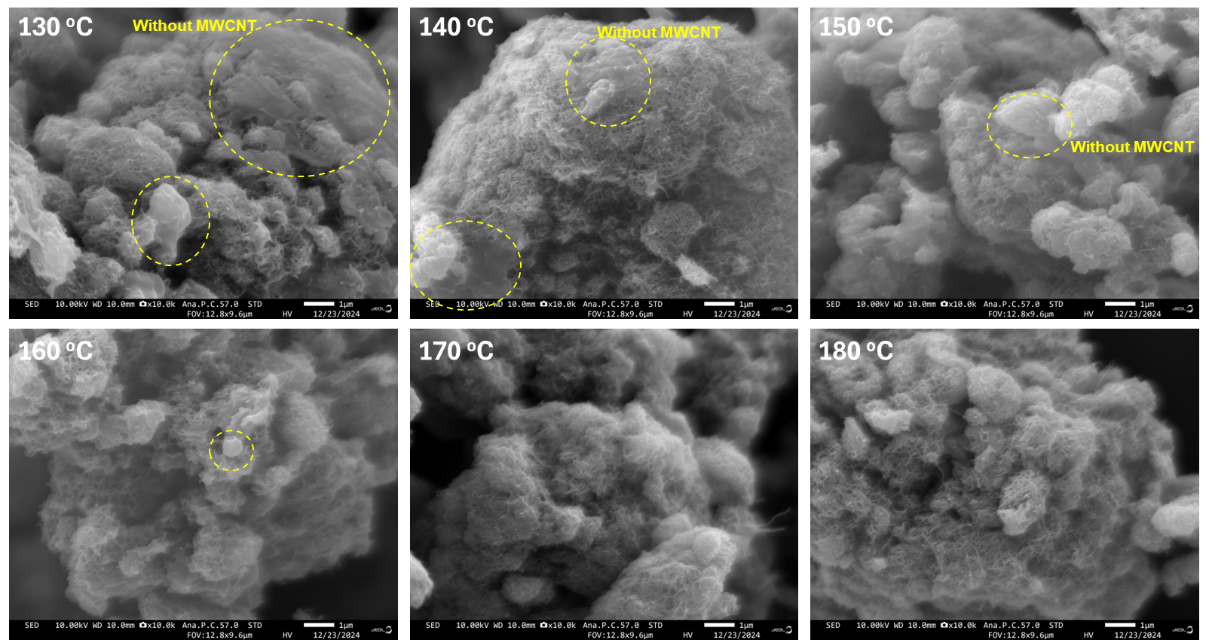


**Figure S13:** The cross-sectional FESEM images of the six fibers subjected to different heat treatment temperatures (130 °C to 180 °C), at a magnification of 10000×.

At 5000× and 10,000× magnifications, MWCNTs become directly observable. In these SEM images, the MWCNTs appear as fuzzy MWCNT@TPU particles on the surface. However, despite the structural similarities among the six samples subjected to different heat treatment temperatures, subtle differences can be identified.

Yellow dashed circular frames highlight certain smooth areas in the images, indicating regions where MWCNTs are absent. These smooth, non-conductive regions are more prominent on the surface of fibers treated at lower temperatures (130°C and 140°C) and can also be observed in **Fig.3c(III)** of the main text. In contrast, such smooth regions are less frequently observed on fibers treated at higher temperatures (above 160°C).


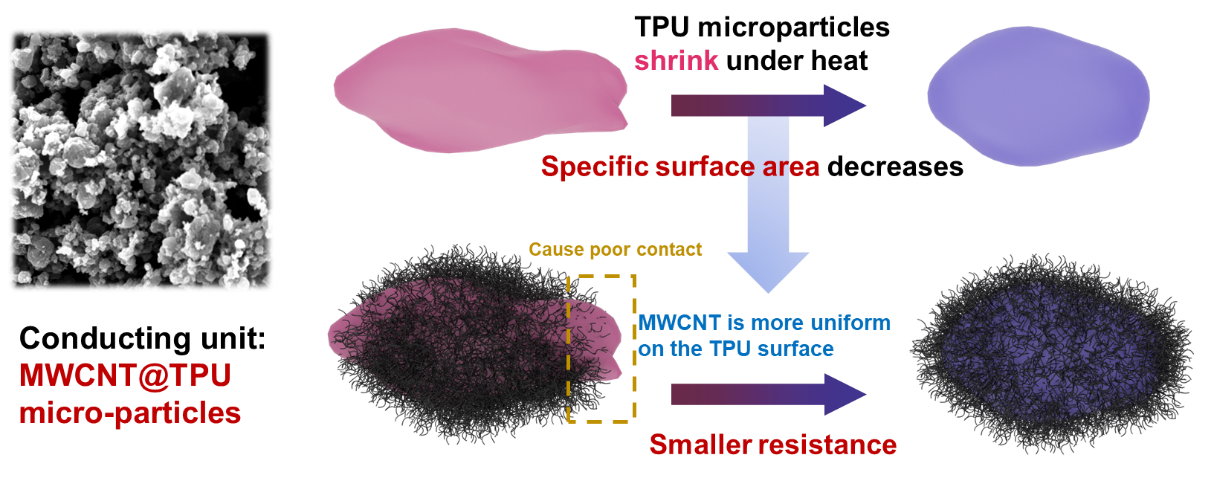


**Figure S14:** A schematic diagram illustrating the mechanism for the further reduction in the resistance of TT/MT fibers.

Based on the SEM image (**Fig.S9-13**), We propose a mechanism explaining the further reduction in the resistance of TT/MT fibers when subjected to heat treatment from 150°C to 170°C. Similar to the reduction in fiber radius caused by the contraction of the outer TPU layer, the MWCNT@TPU particles within the fibers also undergo a degree of shrinkage during the heat treatment. This shrinkage slightly reduces the specific surface area of the internal TPU. However, since the amount of MWCNT remains constant, the contraction of the TPU microparticles enables the MWCNTs to more effectively wrap around the surface of the TPU particles. When the MWCNTs achieve better coverage of the TPU surfaces, the conductive pathways on the surface become more uniform, facilitating electron transfer between the TPU microparticles. In essence, the heat treatment optimizes the conductive pathways, thereby reducing the overall resistance.


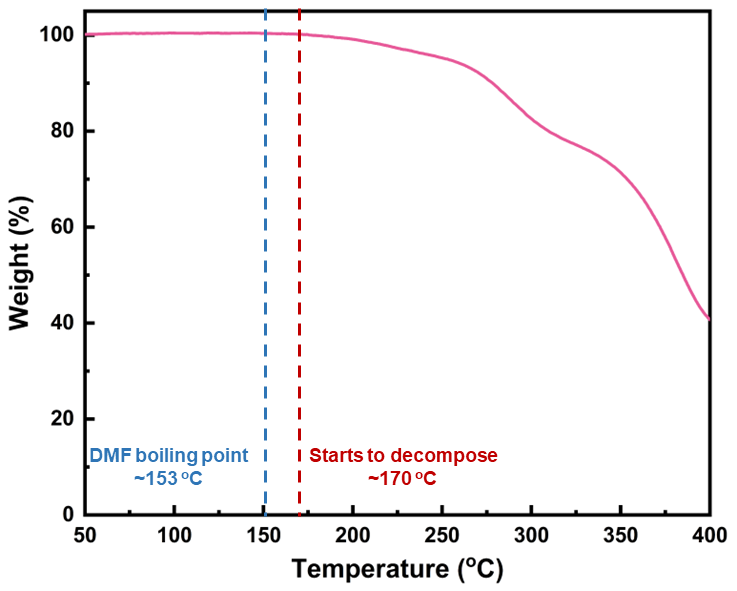


**Figure.S15** The thermogravimetric analysis of fibers.


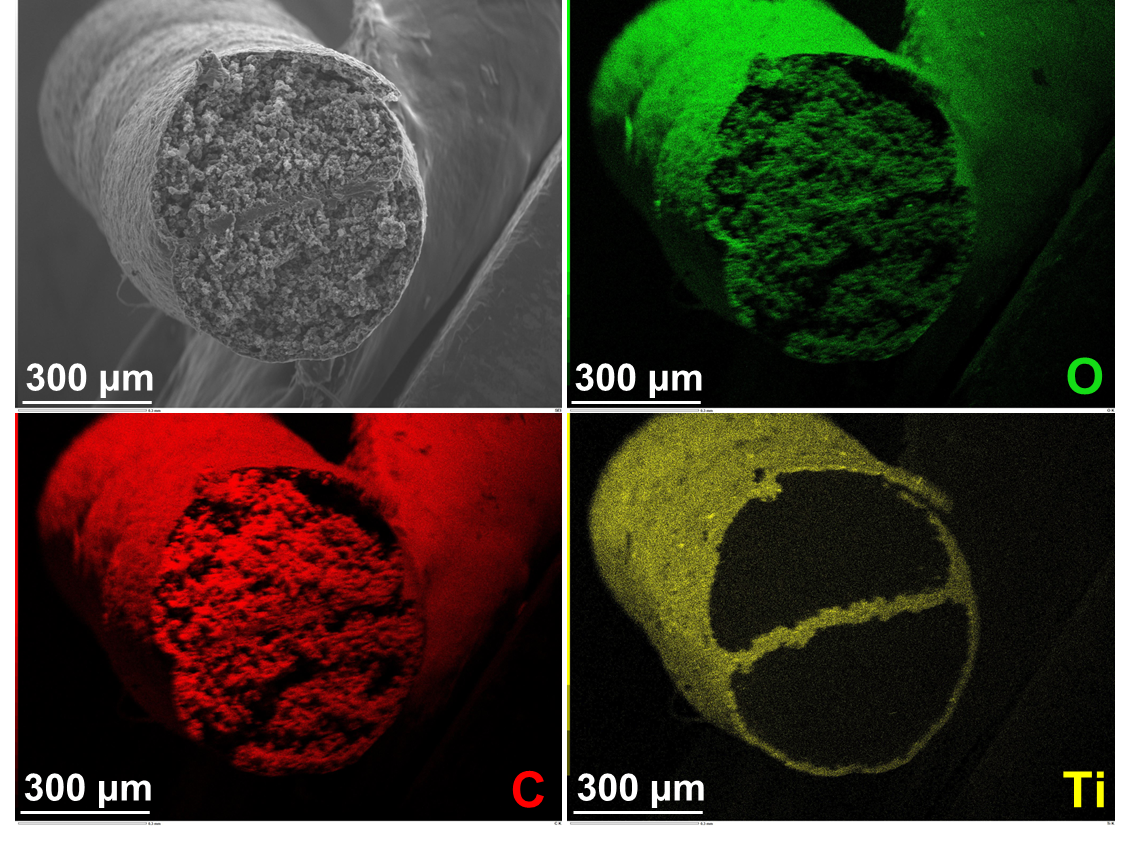


**Figure S16:** EDS map of double-helical TT/MT fiber (sectional view).


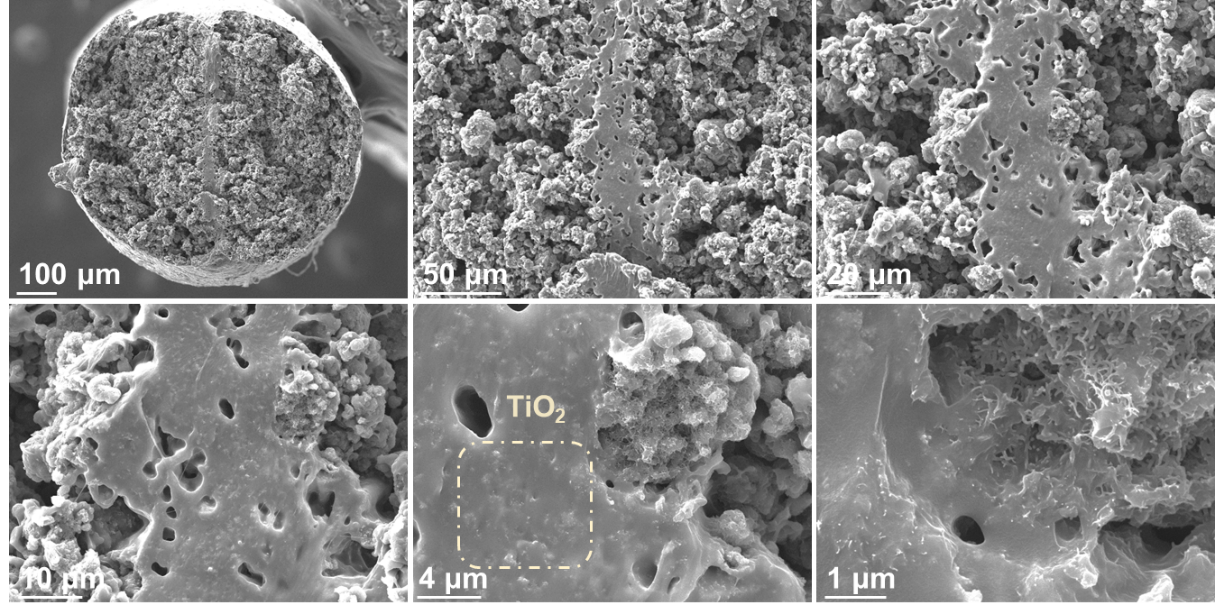


**Figure S17:** More detailed SEM image of double-helical TT/MT fiber (sectional view).

As shown in **Fig.S17**, the TPU-TiO_2_ layer structure in the middle of the double-helical TT/MT fiber remains intact, with complete and thorough adhesion. The entire fiber exhibits a round shape, and the adhesive layer in the center of the fiber is uniform and undamaged.


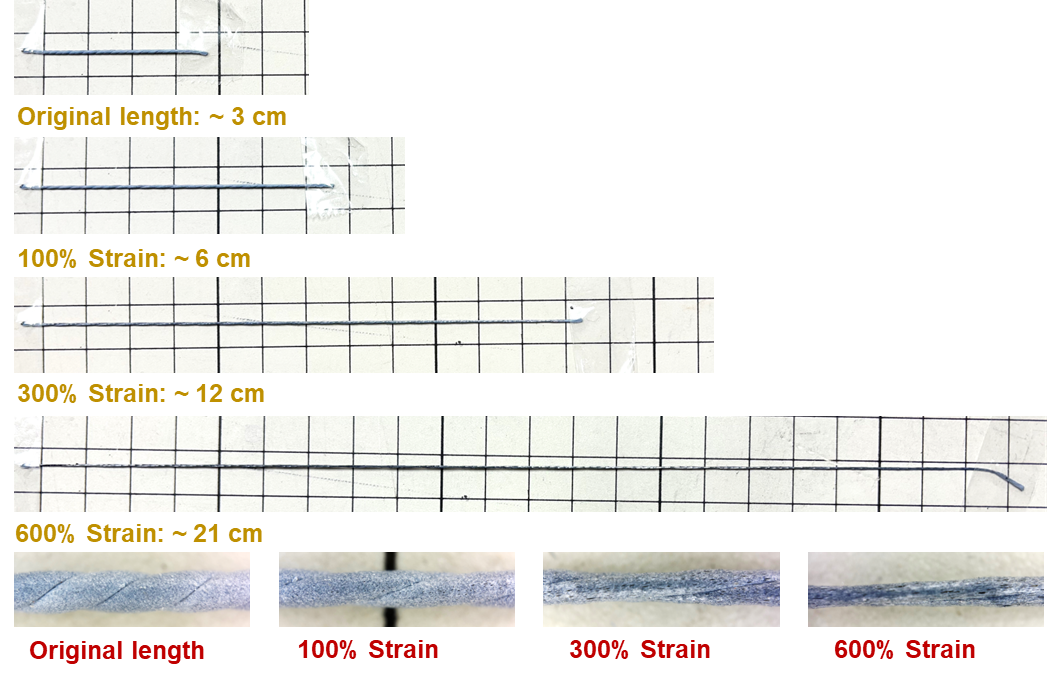


**Figure S18:** Images of double-helical TT/MT fibers in their initial state and under varying degrees of strain

We adhered both ends of the fibers to the table using tape for fixation. It can be observed that under 0–600% stretching, the fibers did not exhibit any unwinding of the helix. The two fibers remained tightly bonded. At 600% stretching, there was slight damage at the points where the fibers were adhered to the tape. This may be attributed to surface damage caused by contact with the tape. However, the fibers as a whole showed no tendency to unwind and retained their double-helical structure, with the two TT/MT fibers remaining firmly connected.

This observation supports the conclusion that the double-helical structure remains intact and does not unwind, a result achieved through appropriate thermal treatment. For TT/MT fibers, fine-tuning the core-shell structure allowed the surface layer to achieve sufficient adhesion at 160 °C (with the soft segments of TPU being activated) while maintaining structural integrity (with the hard segments of TPU remaining stable). In the helical state, internal stress between the two fibers ensured thorough adhesion. Upon cooling, the adhesion strength was retained, preventing the fibers from separating under external forces. Compared to other methods for bonding double-helical fibers, this approach offers significant advantages, making it one of the innovations of this study.


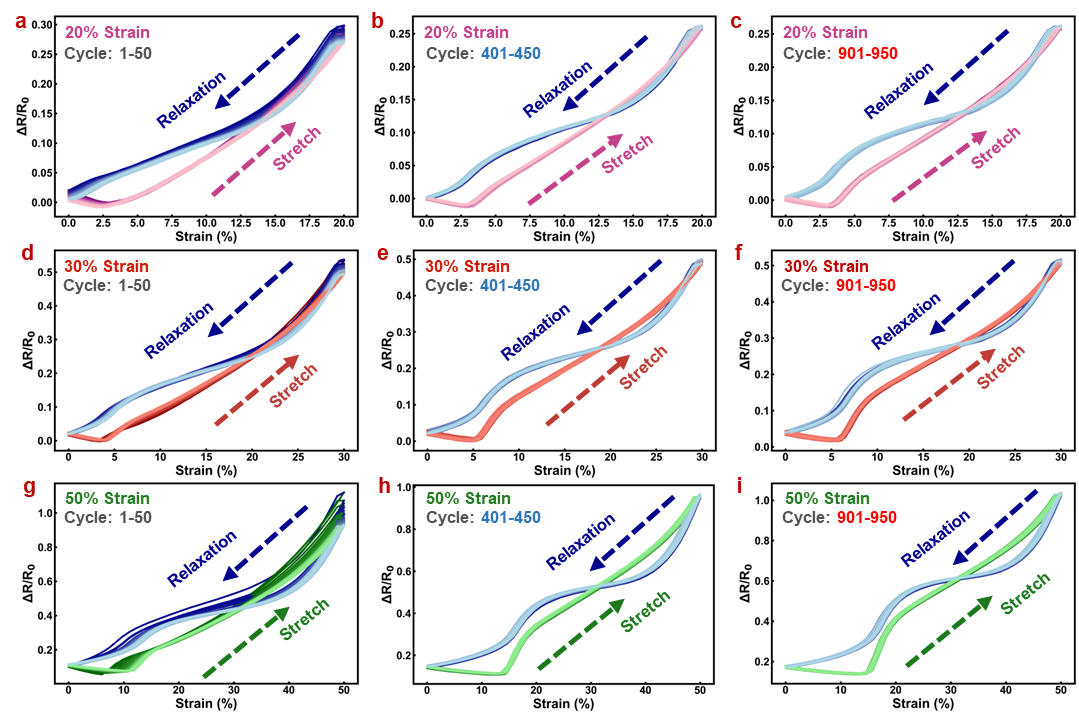


**Fig.S19**: Hysteresis curves of the TT/MT double-helical fiber under different stretching amplitudes. The tested sample has a length of 40 mm, with a stretching speed of 100 mm/min:(a–c) Hysteresis curves for stretching and releasing under 20% strain during cycles 1–50 (a), cycles 401–450 (b), and cycles 901–950 (c); (d–f) Hysteresis curves for stretching and releasing under 30% strain during cycles 1–50 (d), cycles 401–450 (e), and cycles 901–950 (f); (g–i) Hysteresis curves for stretching and releasing under 50% strain during cycles 1–50 (g), cycles 401–450 (h), and cycles 901–950 (i).

As shown in the **Fig.S19**, these are the hysteresis curves of the TT/MT double-helical fiber under different stretching amplitudes. From the data, it can be observed that the TT/MT double-helical fiber sensor exhibits a certain degree of hysteresis. Specifically, under larger stretching amplitudes or after an increased number of cycles (e.g., **Fig.S19(h-i)**), the fiber experiences a slight decrease in resistance during the stretching process. Additionally, a shoulder peak may appear during the release process. This behavior is likely attributable to the inherent properties of the TPU matrix material.

During rapid, high-frequency stretching of TPU/MWCNT flexible composite fibers, the inherent viscoelasticity of TPU prevents it from immediately returning to its original length. This results in a hysteresis effect reflected in the electrical response. At the onset of stretching, the fiber and its internal conductive network (formed by MWCNTs within the TPU matrix)—which may have been bent or not fully aligned—can become straighter, causing a temporary decrease in resistance. In the recovery stage, the brief, rapid return of the resistance value and the subsequent “shoulder peak” may be attributed to the local reconfiguration or redistribution of the MWCNT network after stretching. In other words, as the fiber relaxes, some MWCNT pathways are momentarily reconnected, leading to a rapid reduction in resistance. These phenomena all stem from the viscoelastic nature of TPU and its coupling with the MWCNT conductive network.


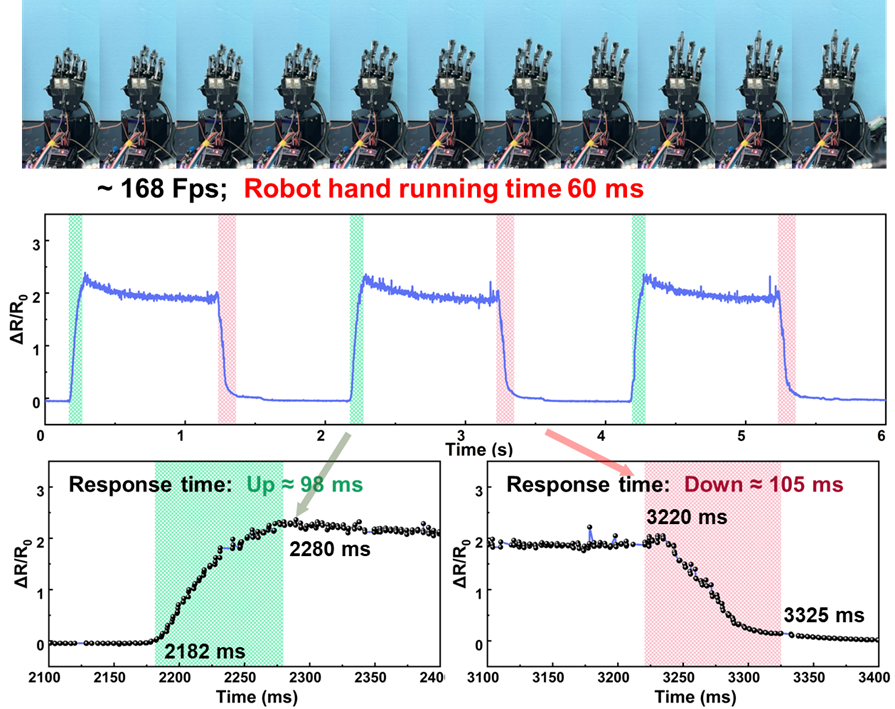


**Figure.S20** Measurement and results of response time of TT/MT double helical senor.

**Testing Method:** As shown in **Fig.S20(a)**, considering the limitations of using a stretching machine (maximum stretching speed of 300 mm/min, requiring at least 6 seconds to stretch 3 cm, which is significantly longer than the sensor's response time) and the inaccuracy of manual stretching, we utilized a robotic hand for more precise response time testing. The robotic hand is equipped with a high-speed motor capable of completing rotational motions within tens of milliseconds. To measure the exact movement time of the robotic hand, we employed high-speed imaging. Using the "slow-motion" feature of the iPhone 15, we recorded the robotic hand's motion. After capturing the video, we exported it as individual frames and calculated the duration based on the actual frame rate of the high-speed video. In this experiment, the iPhone 15 achieved an actual frame rate of 168 fps (although the theoretical maximum frame rate in slow-motion mode is 240 fps, factors such as light exposure can reduce it). The robotic hand took approximately 10 frames to transition from fully closed to fully open, corresponding to a calculated movement time of about 60 ms. To measure the resistance of TT/MT double-helical fibers, we employed a series voltage divider principle, using the A0 interface of an Arduino Uno at a baud rate of 115200 for high-frequency voltage measurements, which were then converted to resistance values. Using this setup, we determined the response time of TT/MT double-helical fibers under big strain.

**Result:** As shown in **Fig.S20(b-c)**, when stretched over a larger range, the fibers exhibited a total response time of approximately 102 ms for resistance increase during stretching and around 105 ms for resistance decrease during recovery.

**Conclusion:** Considering the robotic hand's mechanical movement time of 60 ms, the total response times for TT/MT double-helical fibers under stretching and release are approximately 102 ms and 105 ms, respectively. After accounting for the mechanical stretching duration, the intrinsic response time of the TT/MT double-helical fibers is estimated to be less than 100 ms, making them suitable for applications such as human motion detection and other dynamic scenarios.


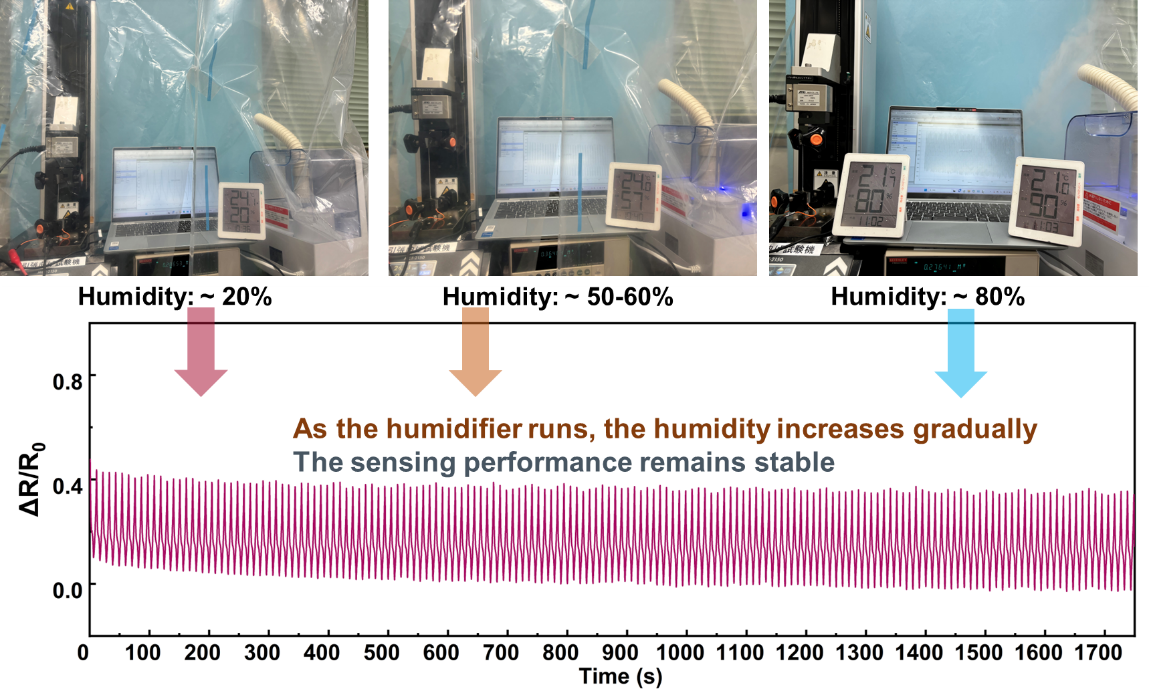


**Figure.S21:** Test results of TT/MT double-helical fibers under different humidity conditions

As shown in **Fig.S21**, we tested the performance of TT/MT double-helical fibers in environments with varying humidity levels. The testing setup involved enclosing the TT/MT flexible sensor with a plastic film, placing a humidifier and a standard household temperature-humidity sensor inside the enclosure. As the humidifier operated, the humidity within the plastic film enclosure rapidly increased from an initial ~20% to a peak of 80-90%. During this process, the TT/MT double-helical fibers remained fully operational.

Based on the recorded data during operation, it was observed that the fibers exhibited stable performance across the entire humidity range of 20-80%. This stability is attributed to the conductive mechanism of TT/MT double-helical fibers, which relies on the internal MWCNT (multi-walled carbon nanotube) conductive network, enabling electronic conduction. Unlike sensors such as hydrogels that utilize ionic conduction, TT/MT double-helical fibers are capable of functioning reliably in extremely dry or highly humid conditions


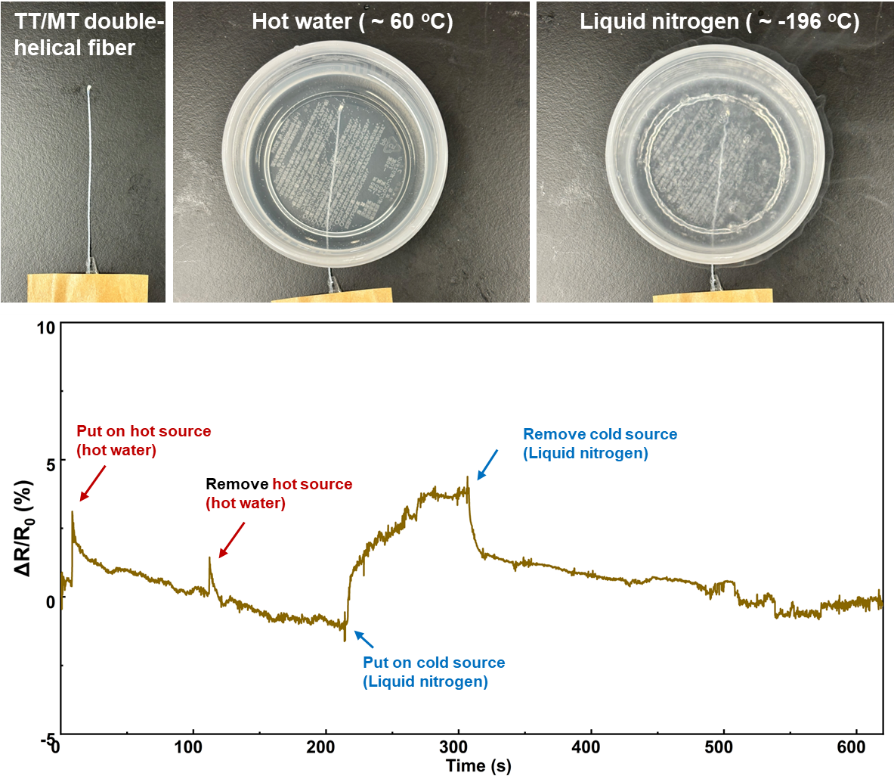


**Figure.S22:** Test results of TT/MT double-helical fibers under different temperature conditions

As shown in **Fig.S22**, we tested the performance of TT/MT double-helical fibers under different temperature conditions. To simulate high-temperature scenarios, we used hot water at 60°C as the heat source, while liquid nitrogen served as the cold source to simulate low-temperature conditions.

When the fibers came into contact with the heat source, a slight decrease in resistance was observed, likely due to increased molecular motion of TPU at higher temperatures, which may have accelerated the recovery of its elastic deformation, leading to reduced resistance. In contrast, upon contact with the cold source, the resistance showed a moderate increase but returned to its original value once the cold source was removed.

In both cases, whether exposed to heat or cold, the change in resistance was minimal (less than 5%). This indicates that the TT/MT double-helical fibers can stably maintain conductivity and function in temperatures ranging from -196°C to 60°C. However, considering the glass transition temperature and cold resistance of TPU, we conclude that TT/MT double-helical fibers can reliably operate within a practical temperature range of about -30 °C to 60 °C, covering conditions found in the vast majority of global environments. Compared to other flexible sensors that use hydrogels, liquid metals or ionic liquids as conductive materials, TT/MT double-helical fibers offer significant advantages.


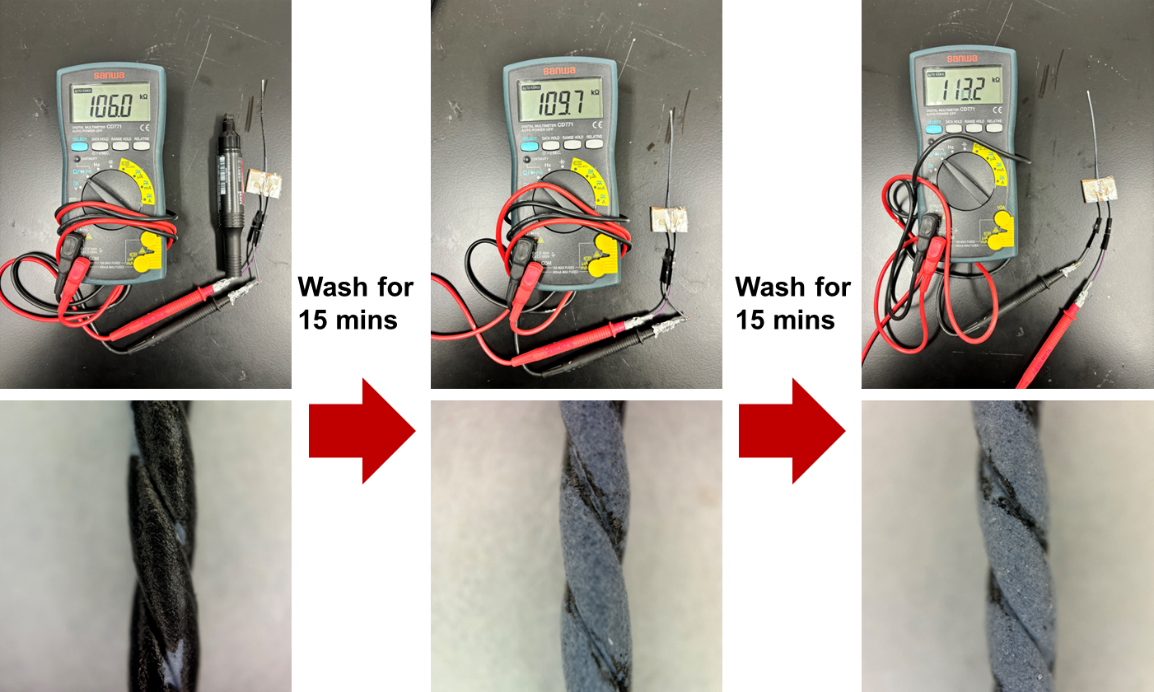


**Figure.S23** The washability performance of TT/MT double-helical fibers

As shown in **Fig.S23**, we tested the washability of TT/MT double-helical fibers under different temperature conditions. To conduct the test, the TT/MT fibers were marked with black ink, and their initial resistance was measured to be approximately 106 kΩ. After washing the fibers for 15 minutes, the resistance increased slightly to 109.7 kΩ. A subsequent 15-minute wash resulted in a resistance of 113.2 kΩ.

These results demonstrate that TT/MT double-helical fibers are washable to a certain extent. While the resistance slightly increases after washing, this is primarily due to reversible elastic deformation, which allows for partial recovery over time. Additionally, the fibers are protected by a dense surface layer, and the TPU material is inherently hydrophobic. This ensures that short-term washing does not allow water to penetrate the fibers, preserving their structural and electrical integrity.


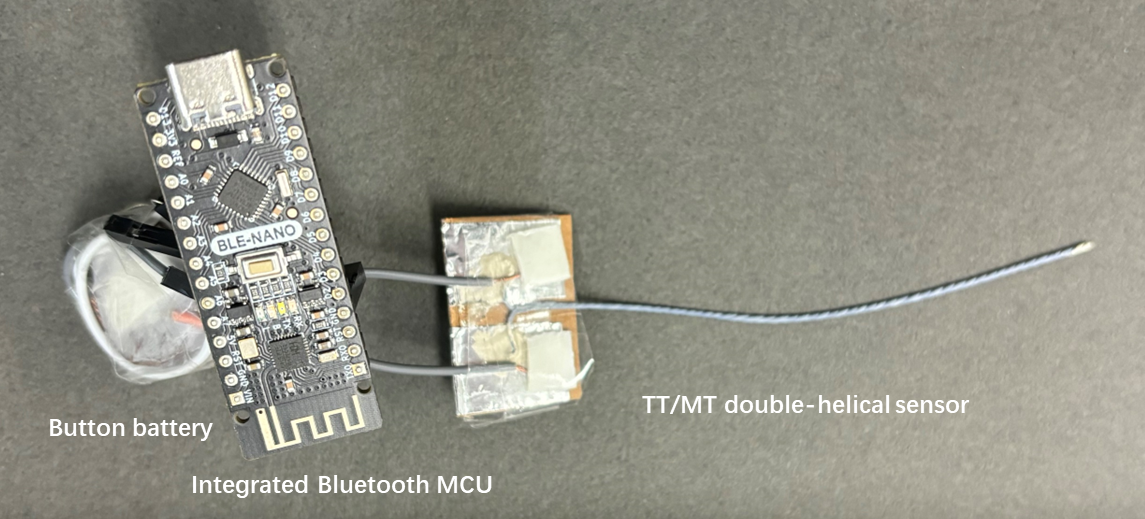


**Figure S24**: Schematic diagram of the structure for remote sensing using the TT/MT double-helical fiber

As shown in **Fig.S24**, this is a schematic diagram of the structure for remote sensing using the TT/MT double-helical fiber. The setup includes an Arduino-compatible microcontroller integrated with a Bluetooth chip, two 3 V button batteries, and the TT/MT double-helical fiber. The measurement of resistance changes is achieved through a series voltage divider method, similar in principle to the smart glove shown in **Fig.8** of the main text. Related videos demonstrating the use of this setup can be found in **Movies S3-S4**.


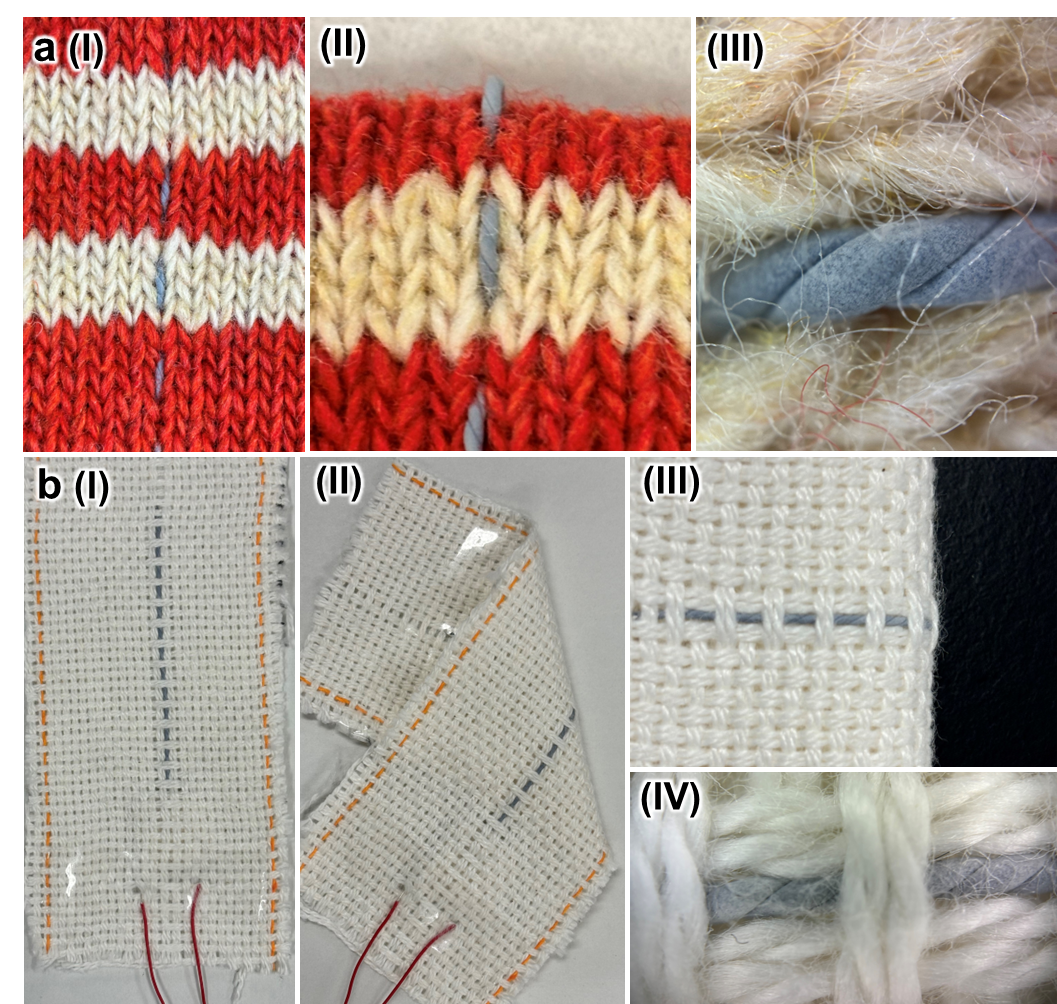


**Figure.S25** The photo of TT/MT double-helical fibers embedded in knitted (a) and woven (b) fabrics

As shown in **Fig.S25**, we successfully integrated TT/MT double-helical fibers into commonly used knitted and woven fabrics, demonstrating that these fibers are indeed weavable. This indicates their potential for embedding into textiles to create smart fabrics in the future. However, challenges arise during the integration process due to the mismatch between the modulus of the fibers in these fabrics and that of the TT/MT double-helical fibers. The fibers in common fabrics are nearly inelastic, which hinders the deformation of the TT/MT double-helical fibers to some extent and reduces their performance. Additionally, when embedding TT/MT double-helical fibers into these fabrics, we opted for looser fiber structures to minimize excessive friction that could impose stress on the fibers, thereby avoiding performance degradation. However, this loose structure can lead to the fibers detaching from the fabric during application.

In the future, we plan to collaborate with manufacturers to develop fibers with modulus and properties that align with the TT/MT double-helical fibers, such as elastic fibers capable of deformation under tension. By employing woven fabric manufacturing techniques, TT/MT double-helical fibers can replace one of the warp or weft threads during the production process, embedding them tightly into the inner structure of the fabric. Unlike post-production forced integration, this method ensures that the fibers are securely fixed by friction during production while avoiding performance degradation caused by external forces. This approach would enable the mass production of smart fabrics that are low-cost, comfortable to wear, highly durable, and exhibit excellent performance. Such smart fabrics could be widely applied across various fields.


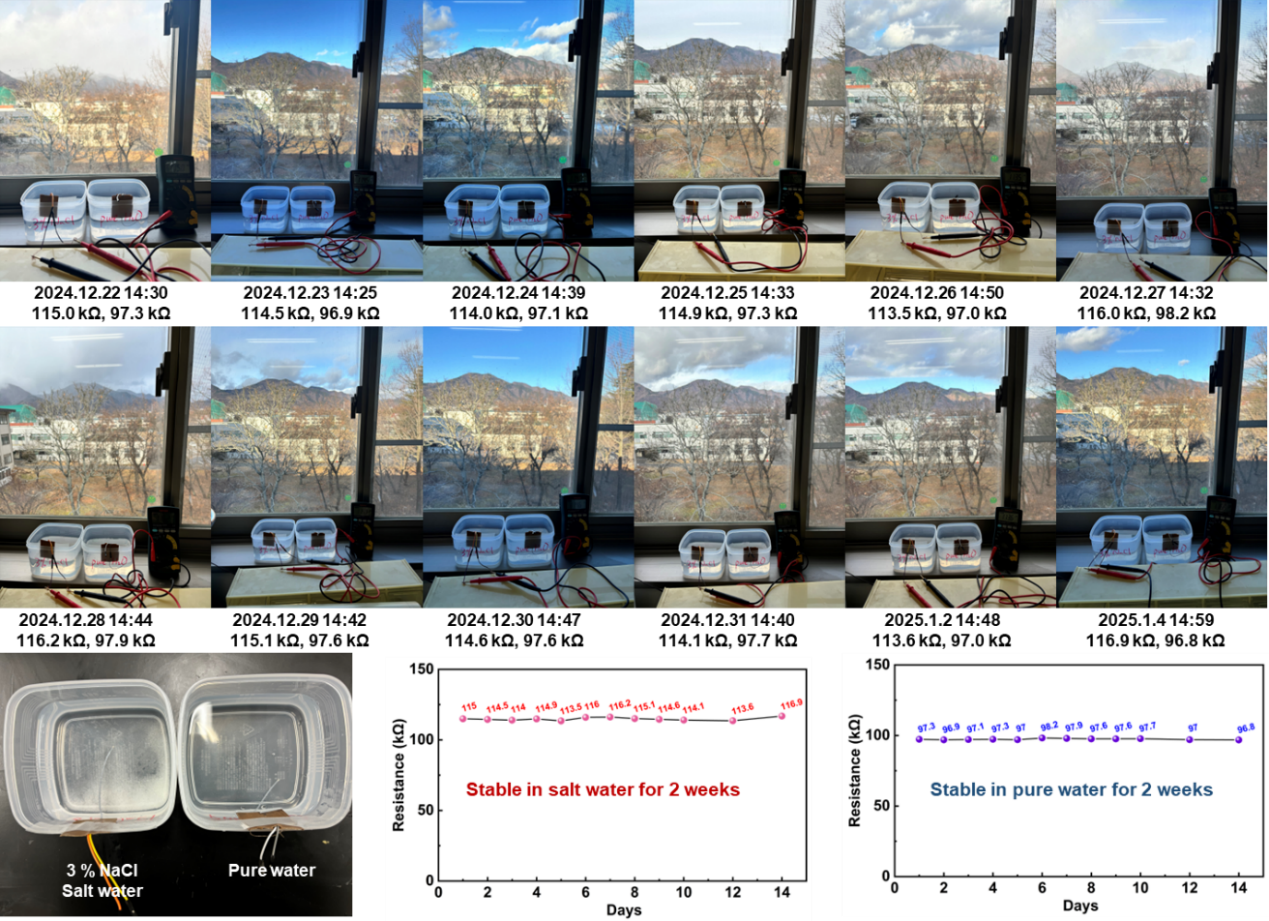


**Figure S26** Water resistance performance of the TT/MT double-helical fiber sensor after waterproof treatment.

As shown in **Fig.S26**, we conducted daily resistance measurements of the TT/MT double-helical fiber sensor immersed in a 3% sodium chloride solution (left) and pure water (right) at approximately 2:30 PM (Japan time) each day from ***December 22, 2024***, to ***January 4, 2025***. The results indicate that the resistance of the sensor in the 3% sodium chloride solution ranged from 113.5 kΩ to 116.9 kΩ (a 2.9% variation), while the resistance of the sensor in pure water ranged from 96.9 kΩ to 98.2 kΩ (a 1.3% variation).

No significant resistance changes were observed. If a short circuit or damage had occurred, the resistance would have either continuously decreased significantly (short circuit, due to saltwater entering the fiber) or increased markedly (damage, due to water disrupting the internal conductive network continuity). Based on these results, we conclude that the TT/MT double-helical fiber sensor remains stable and operational for at least 14 days.


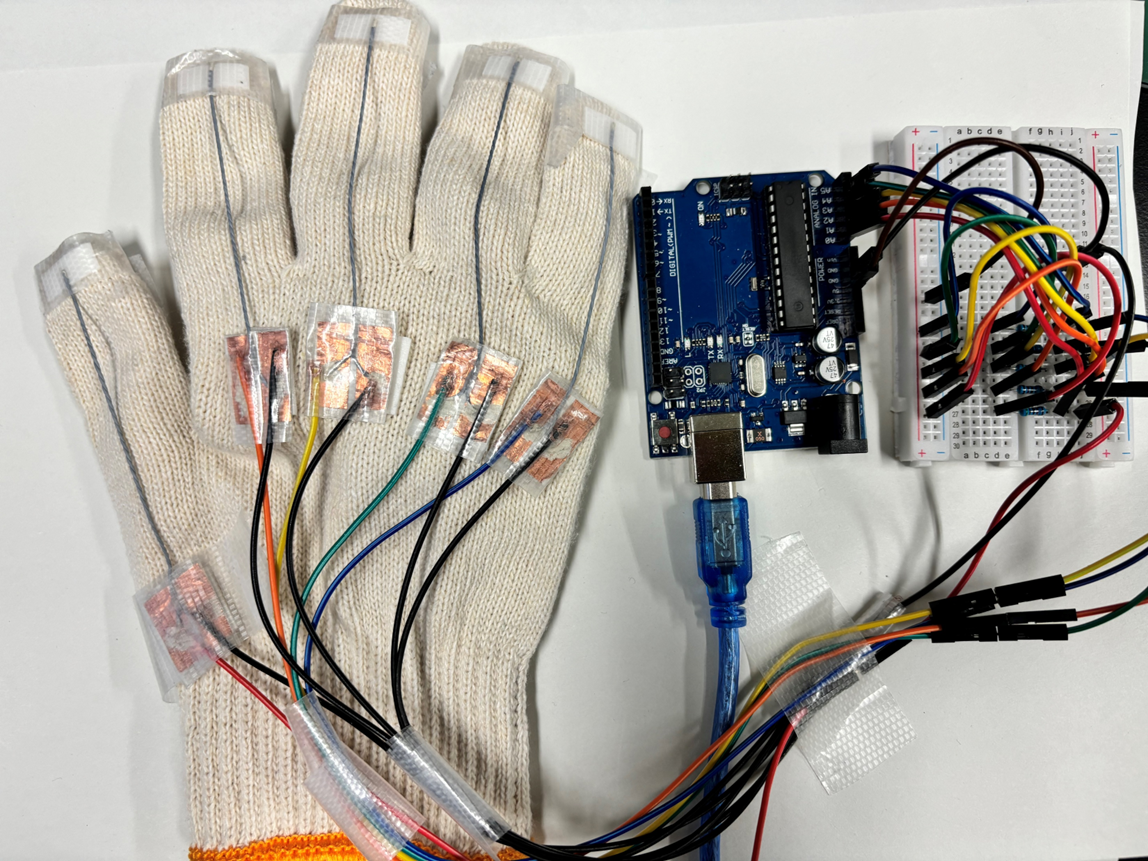


**Figure S27:** Photo of the entire smart glove device.


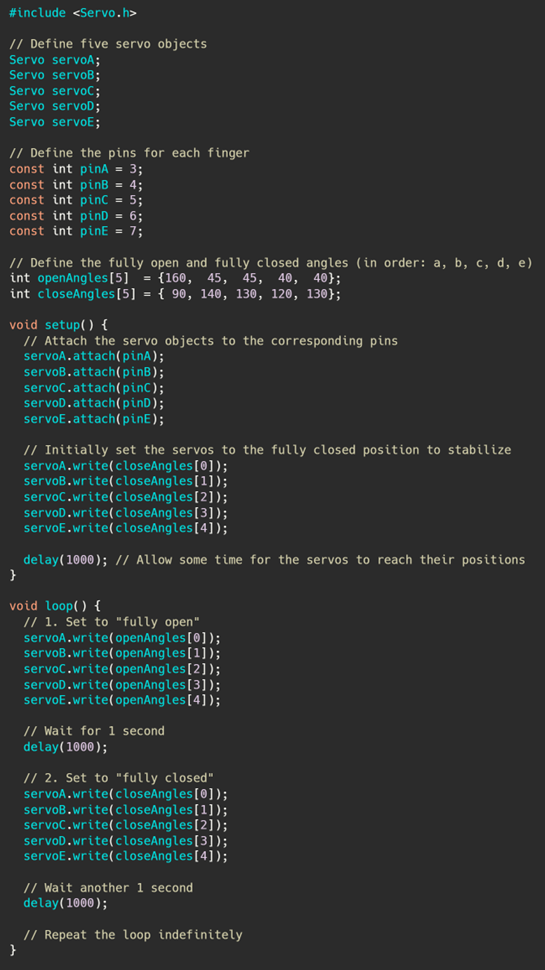


**Figure S28** Code related to using the Arduino microcontroller to control the manipulator


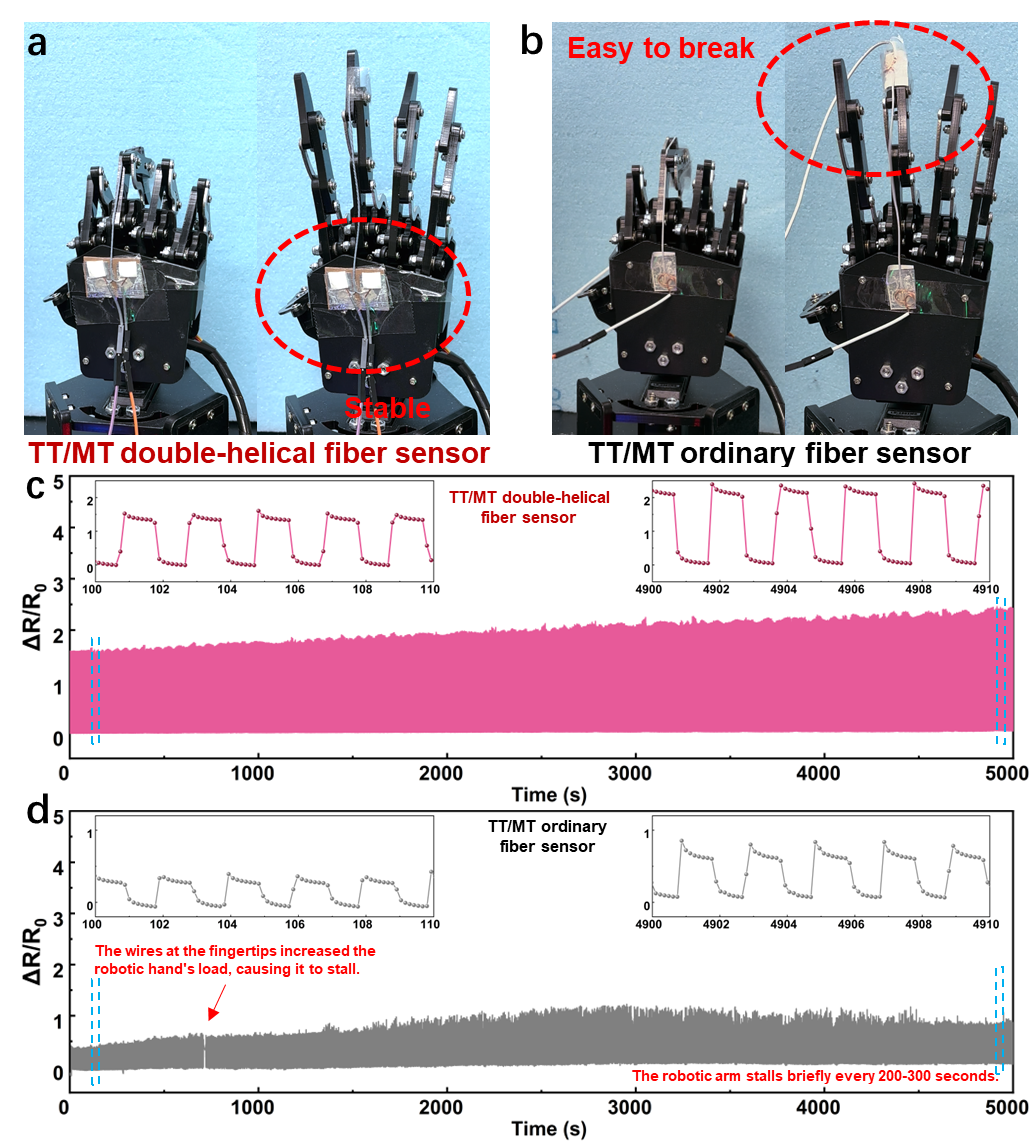


**Figure.S29.** Comparison of performance in practical sensing applications between TT/MT double-helical fibers and ordinary fibers (TT/MT fibers without twist treatment or heat treatment) using a robotic hand. **(a)** Photographs showing two states of the robotic hand using TT/MT double-helical fibers as sensors. **(b)** Photographs showing two states of the robotic hand using ordinary fibers as sensors. **(c)** Sensing signals recorded over 5000 seconds of program operation using TT/MT double-helical fibers as sensors **(d)** Sensing signals recorded over 5000 seconds of program operation using ordinary fibers as sensors.

As shown in **Fig.S29(a)**, when using TT/MT double-helical fibers, both electrodes of the fiber are attached to the back of the robotic hand, which remains relatively stationary during operation. No electrodes are placed on the robot's fingertip, preventing wires from being pulled during movement. In contrast, as shown in **Fig.S29(b)**, for ordinary fiber sensors with electrodes on both ends, one electrode is installed on the fingertip of the robotic hand. This setup forces the robotic hand to drag the wires during motion, **increasing resistance to movement and posing a risk of damaging the sensor**. Similarly, in practical human applications, traditional fiber-based flexible sensors often encounter issues such as electrode damage or discomfort caused by excessive movement resistance.

From the comparison between **Fig.S29(a)** and **Fig.S29(b)**, it is evident that the smaller diameter of TT/MT double-helical fibers helps reduce the burden on the wearer in practical applications. Additionally, a 5000-second cyclic test (~2500 cycles) was conducted. While slight inaccuracies may exist due to the Arduino Uno microcontroller's CPU timing errors, and minor stalling of the robotic hand may occur every 250 seconds due to equipment limitations, these factors do not significantly affect the results. The testing outcomes for TT/MT double-helical fibers and ordinary fibers are shown in **Fig.S29(c)** and **Fig.S29(d)**, respectively.

When using TT/MT double-helical fibers, the resistance change rate signal is relatively larger. This is likely because the twisting and heat treatment of TT/MT fibers may have altered their internal conductive network, making it denser. The twisting also acts as a pre-stretching process, enhancing the sensitivity coefficient of the fiber. More importantly, the sensing signal of the TT/MT double-helical fiber is significantly more stable, a crucial aspect for flexible sensors and electronic devices. Since the TT/MT fiber has no electrodes at the fingertip, it avoids damage caused by the pulling of electrodes during movement. In contrast, the signal from traditional fibers is less stable, possibly due to prolonged pulling of the electrodes and the obstruction caused by the wires, which add substantial load to the servo motor, leading to unstable operation and occasional stalling.

In summary, double-helical fiber-based flexible sensors with same-side electrodes demonstrate significant advantages in sensitivity, signal stability, and comfort during joint applications, as they do not impose additional burdens on joints.


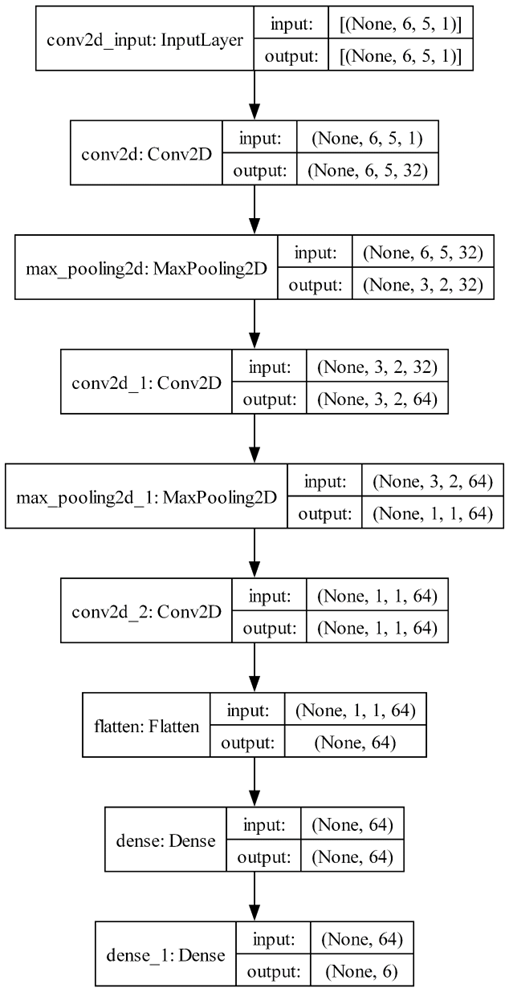


**Figure S30:** Flowchart of CNN machine learning steps for gesture recognition.


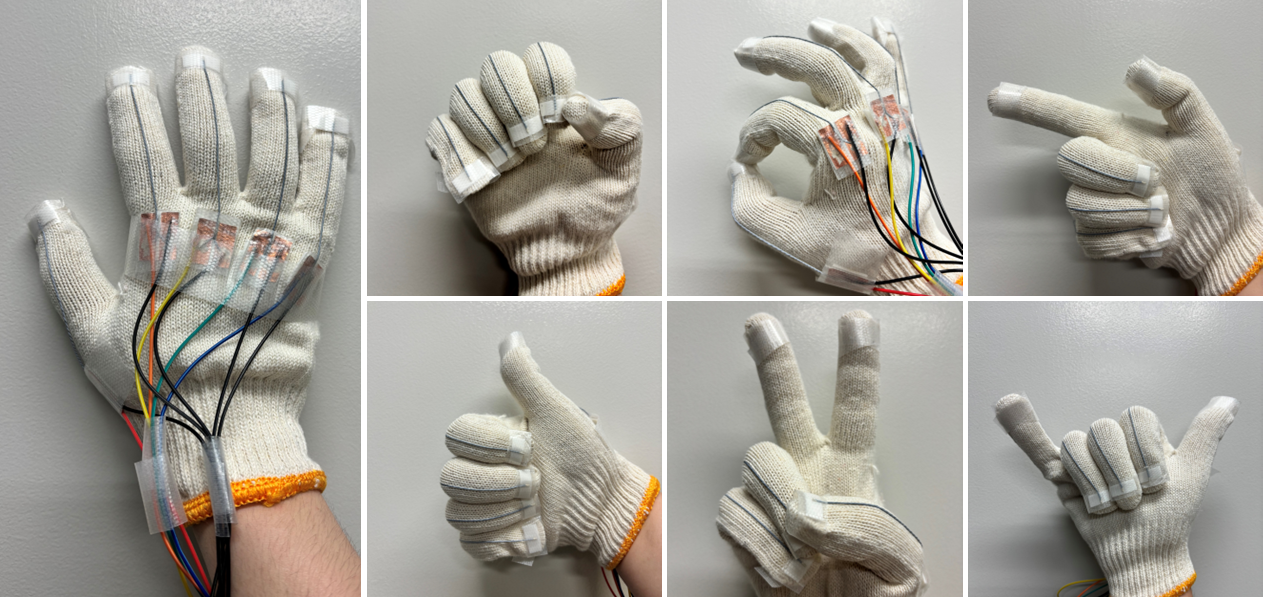


**Figure S31:** Photos of six common gestures while wearing smart gloves made of TT/MT double-helical fibers.

As shown in **Fig.S31**, these are photos of six common hand gestures while wearing TT/MT fibers, provided for reference.

**Table S1** Comparison of related research work in the past year

| Appearance of The Fiber | | Preparation Methods | | Sensitivity and Performance | Applications | Ref.  No. |
| --- | --- | --- | --- | --- | --- | --- |
| Diameter | Structure | Materials | Techniques |  |  |  |
| < **0.7 mm** | Double-helical | TPU, MWCNT, TiO_2_ | Coaxial Wet-spinning + Twist and Heat treatment | **GF=0.9-349**  >300% working range  Unique same-side dual electrodes design | Remote motion detection, Gesture recognition, etc. | **This work** |
| **≈ 1.5 mm** | Double-helical | Liquid Metal, SEBS | Thermal Drawing and Spiraling | **GF=0.1917-12.855**  >1000% stretchability Eliminates directional errors. | Wearable electronics, Human motion detection, etc. | **[16]** |
| **≈ 0.8 mm** | Helical | TPU, MXene, CNT, PDMS | Electrospin + Rolling and Twisting | **GF=0.43-17.8**  Strain range up to 700%  High stability | Monitoring hand rehabilitation, Wearable electronics, etc. | **[34]** |
| **≈ 1.2 mm** | Double-helical | CNT, Ecoflex Mandrel Fiber | Twisted and Stretched Fiber Wrapped with CNT ribbons | **Multifunctional**  Torsion of ±2520 rad/m, Sensing of 38 kPa | Capacitive sensor  supercapacitor, tensile actuator, etc. | **[43]** |
| **≈ 0.9 mm** | Embedded | CuI, PU | Solution-based Chemical Synthesis Method | **GF=0.36-3.89**  High stretchability  Seebeck coefficient of ≈203.6 μV/K | Detection of temperature, strain, pressure, etc. | **[44]** |
| **≈ 1 mm** | Hollow | AgNPs,  TPU, PVA | Cryo-spun Drying Strategy | **GF=4.68**  Strength of 3.69 MPa  wide-range linearity (R^2^ = 0.983) | Wearable strain sensors, monitor human motion, etc. | **[45]** |
| **≈ 0.5 mm** | Multilayer | TPU, Galinstan, Nickel particles | Layer by layer coating | **Capacitive sensor**  High stretchability and conductivity (4537.28 S/cm). | Healthcare monitoring, detect physiological signals, etc. | **[46]** |

To highlight the contributions and innovations of this study, we compared our work on TT/MT double-helical fiber-shaped sensors with six key papers published in 2024 on fiber-shaped flexible sensors (DOIs: 10.1016/j.jmst.2024.02.028, 10.1021/acssensors.4c00164, 10.1002/adfm.202312033, 10.1002/adfm.202407759, 10.1007/s42765-024-00460-2, 10.1002/advs.202412859). The comparison is organized across three aspects: fiber dimensions, fiber structure and fabrication method, and fiber performance and applications.

**1. Fiber Dimensions**

The TT/MT double-helical fiber sensor developed in this study exhibits significantly smaller dimensions compared to other double-helical fiber sensors **[16][43]**. Its diameter is less than 0.7 mm, which is notably smaller than other fiber-shaped sensors with different structures **[34][44][45]**. The cross-sectional area of fibers is proportional to the square of the radius. For instance, fibers with a diameter of 1.2–1.5 mm have cross-sectional areas 3–4 times larger than our TT/MT fiber, representing a substantial improvement. A smaller cross-sectional area translates into advantages in terms of cost, comfort, and usability.

This improvement is primarily attributed to the innovative fabrication method in this study. By employing a spinning technique with different core (3 mL/h) and shell (0.75 mL/h) speeds, we achieved a thin yet robust outer layer and an ultra-porous inner core. Additionally, incorporating nano-TiO_2_ into the fiber enabled the outer layer to bond effectively during thermal treatment at 160 °C without damage. This bonding strategy ensures that the double-helical structure achieves self-encapsulation, forming nearly circular cross-sections with an overall diameter (~0.69 mm) smaller than the precursor wet-spun fibers (~0.79 mm). This innovation resolves the challenge of oversized double-helical fiber structures, making their dimensions comparable to other fiber sensors.

**2. Fiber Structure and Fabrication Process**

The most significant structural advantage of the double-helical design in this study is the realization of single-sided dual-electrode sensor assembly. In this fiber, one side contains two electrodes while the other side remains electrode-free. This unique design prevents damage caused by electrode stretching during joint movement. It allows the electrode-free side to be placed in highly dynamic regions while positioning the dual-electrode side in relatively static areas, protecting the electrodes from mechanical strain.

In terms of fabrication, this study presents a more convenient approach than other double-helical fabrication methods. For instance, ***Zhang et al***. used a thermal stretching and helical design process to create double-helical fibers, leveraging SEBS polymer elasticity and liquid metal conductivity, but required thick external encapsulation layers to prevent leakage **[16]**. Similarly, ***Son et al.*** twisted and pre-strained rubber fibers, applied CNT strips, and relied on mismatch deformation to generate the helical structure **[43]**. In contrast, our method, combining coaxial wet spinning with thermal treatment for inner encapsulation, is simpler, reduces cross-sectional area by over 50%, and ensures a higher volume fraction of conductive material.

Notably, the volume of conductive material in our fibers exceeds 90% (as seen in **Fig.5i** and **Fig.5j**), compared to approximately 20% in ***Zhang et al.'s*** fibers **[16]** or less in ***Son et al.'s*** surface-deposited designs **[43]**. The high conductive material fraction in our fibers enables superior sensing performance even with a smaller fiber diameter.

This study employed readily available materials such as MWCNT, TiO_2_, and TPU, which are cost-effective and simple to process (MWCNT is industrially available by 2024). In contrast, materials like MXene **[34]** and AgNPs **[45]** are expensive, and liquid metals **[16][46]** carry risks of leakage. Our approach thus offers advantages in material accessibility, cost, and safety.

**3. Fiber Performance and Applications**

The TT/MT fibers exhibit a gauge factor of 0.9–349, comparable to or higher than similar fiber sensors **[16][34][44][45]**. The fibers maintain stable performance after 1,000 cycles of 20–50% tensile strain. Additionally, the natural formation of dual-electrode connections during the twisting process simplifies electrode assembly. The single-sided dual-electrode design offers unique advantages in applications such as joint motion monitoring. For instance, when applied to gesture recognition in smart gloves, all electrodes can be positioned on the back of the hand, ensuring that finger movements do not stress the electrodes. This is a significant improvement over traditional designs where electrodes on fingertips risk damage during rapid motion or accidental impacts.

**Conclusion**

Compared to other fiber-shaped electronic devices published in 2024, our work achieves comparable or slightly superior sensor sensitivity. Additionally, through the innovative design of a same-side dual-electrode double-helical structure, we address the critical issue of electrode damage in flexible sensors caused by joint movement. Furthermore, compared to other double-helical flexible sensors, our fiber diameter has been greatly reduced, while the conductive material volume fraction has been increased to approximately 90%.

[16] Y. Zhang, Y. He, L. Niu, X. Xing, Y. Jiang, J. Fang, Y. Liu, *Journal of Materials Science & Technology* **2024**, *195*, 136-145.

[34] L. Liu, T. Luo, X. Kuang, X. Wan, X. Liang, G. Jiang, H. Cong, H. He, *ACS Sens* **2024**, *9*, 2476-2487.

[43] W. Son, J.M. Lee, J.H. Choi, J. Kim, J. Noh, M. Oh, H.J. Sim, C.K. Jeong, S. Chun, S.J. Kim, C. Choi, *Adv. Funct. Mater.* **2024**, *34*, 202312033.

[44] K. Yoon, S. Lee, C. Kwon, C. Won, S. Cho, S. Lee, M. Lee, J. Lee, H. Lee, K.I. Jang, B. Kim, T. Lee, *Adv. Funct. Mater.* **2024**, *35*, 202407759.

[45] A. Zheng, K. Wan, Y. Huang, Y. Ma, T. Ding, Y. Zheng, Z. Chen, Q. Feng, Z. Du, *Adv. Fiber Mater.* **2024**, *6*, 1898-1909.

[46] J. Liu, Y. Yang, G. Chen, H. Sun, X. Xie, Y. Hou, L. Zhang, J. Wang, J. Wang, *Adv. Sci.* **2024**, 2412859.
